# Elucidating vaccine efficacy using a correlate of protection, demographics, and logistic regression

**DOI:** 10.1186/s12874-024-02197-3

**Published:** 2024-04-30

**Authors:** Julie Dudášová, Zdeněk Valenta, Jeffrey R. Sachs

**Affiliations:** 1 Quantitative Pharmacology and Pharmacometrics, MSD Czech Republic, Svornosti 3321/2, 150 00 Prague 5, Prague, Czech Republic; 2 First Faculty of Medicine, Charles University, Prague, Czech Republic; 3 Statistical Modelling, Institute of Computer Science of the Czech Academy of Sciences, Prague, Czech Republic; 4 Quantitative Pharmacology and Pharmacometrics, Merck & Co., Inc, Rahway, NJ USA

**Keywords:** Correlate of protection, Vaccine efficacy, Relative risk, Baseline covariates, Logistic regression

## Abstract

**Background:**

Vaccine efficacy (VE) assessed in a randomized controlled clinical trial can be affected by demographic, clinical, and other subject-specific characteristics evaluated as baseline covariates. Understanding the effect of covariates on efficacy is key to decisions by vaccine developers and public health authorities.

**Methods:**

This work evaluates the impact of including correlate of protection (CoP) data in logistic regression on its performance in identifying statistically and clinically significant covariates in settings typical for a vaccine phase 3 trial. The proposed approach uses CoP data and covariate data as predictors of clinical outcome (diseased versus non-diseased) and is compared to logistic regression (without CoP data) to relate vaccination status and covariate data to clinical outcome.

**Results:**

Clinical trial simulations, in which the true relationship between CoP data and clinical outcome probability is a sigmoid function, show that use of CoP data increases the positive predictive value for detection of a covariate effect. If the true relationship is characterized by a decreasing convex function, use of CoP data does not substantially change positive or negative predictive value. In either scenario, vaccine efficacy is estimated more precisely (i.e., confidence intervals are narrower) in covariate-defined subgroups if CoP data are used, implying that using CoP data increases the ability to determine clinical significance of baseline covariate effects on efficacy.

**Conclusions:**

This study proposes and evaluates a novel approach for assessing baseline demographic covariates potentially affecting VE. Results show that the proposed approach can sensitively and specifically identify potentially important covariates and provides a method for evaluating their likely clinical significance in terms of predicted impact on vaccine efficacy. It shows further that inclusion of CoP data can enable more precise VE estimation, thus enhancing study power and/or efficiency and providing even better information to support health policy and development decisions.

**Supplementary Information:**

The online version contains supplementary material available at 10.1186/s12874-024-02197-3.

## Background

This work introduces a novel use of immune response biomarkers to help identify baseline covariates affecting vaccine efficacy (VE). VE is defined as a proportional reduction in risk of disease occurrence for vaccinated subjects, compared to placebo control subjects, and is often assessed by counting disease cases and non-cases in randomized controlled clinical trials [1]. Baseline covariates refer to demographic and clinical characteristics (e.g., age, gender, race and ethnicity, or pre-vaccination serostatus) or other information (e.g., time and site of enrollment) collected from subjects before the time of randomization (i.e., random assignment to active vaccination *versus* placebo arm). A randomized controlled trial can be used to estimate VE even if the primary analysis does not consider baseline covariates because, due to randomization, measured and unmeasured covariates will, on average, be balanced between the vaccinated and control groups. However, VE may be affected by baseline covariates (for example, it can vary with age) and understanding the impact of covariates on VE is key to making informed decisions, not only in the development of safe and effective vaccines, but also in public health considerations post-licensure.

Statistical significance of covariate effects on binary clinical outcome (e.g., diseased versus non-diseased) is typically [2–5] evaluated using multiple (also referred to as “multivariable”) logistic regression, which (as used here) involves incorporating multiple explanatory variables (predictors) in the regression analysis for prediction of a single binary outcome. Clinical significance of a covariate effect on VE, a notion different from that of statistical significance, can be assessed by comparing estimated relative risks (RR) between the vaccinated and control subjects in covariate-defined subgroups. Estimated effect can be large in magnitude but not prove statistically significant (e.g., due to variability, trial size, etc.), or can prove highly significant statistically, yet not clinically (e.g., when the effect size is too small to have a measurable impact on public health).

Efficacy trials often measure subjects’ immune response post-vaccination (immunogenicity) as an exploratory endpoint in addition to assessing the trial’s primary clinical endpoint(s). An immunogenicity biomarker which reliably predicts protection is termed correlate of protection (CoP) [6]. The first formal method to validate an immunogenicity biomarker as a CoP using data from a phase 3 trial was proposed by Prentice [7], who introduced the following criteria:

- *Criterion of vaccine efficacy:* demonstrating vaccine effect on the clinical endpoint (e.g., occurrence of disease or infection) evaluated using case-counting
- *Criterion of vaccine immunogenicity:* demonstrating vaccine effect on the immunogenicity biomarker
- *Criterion of a correlate of risk (CoR):* demonstrating that the immunogenicity biomarker correlates with the clinical endpoint
- *Criterion of a CoP:* demonstrating that the probability of the clinical endpoint is conditionally independent of vaccination status when conditioned on the immunogenicity biomarker (indicating that the full vaccine effect is mediated by immunogenicity)

The Prentice framework has gained widespread adoption [8–11] due to its simplicity. In this work, the term “CoP” is used for a biomarker that meets all four Prentice criteria [7] and fully mediates the vaccine effect. It has been shown that CoP-based VE prediction is more precise than case-count-based VE estimation [12].

The work presented here is motivated by the need to assess a natural extension of that result: inclusion of CoP data could increase efficiency in finding covariate effects and comparing VE between subgroups (by reducing the width of CoP-based confidence interval compared to case-counting). VE can be affected in three ways (given immunogenicity is a CoP): either (i) immunogenicity measurements are distributed significantly differently across sub-populations, or (ii) the sub-populations differ in the relationship between immunogenicity and clinical outcome, or (iii) they differ in both the immunogenicity distributions and the relationship. Because the assessment of (i) is typically done using existing methods [13], the work here focuses on the harder problem of assessing (ii) and (iii).

The aim of this work is to compare two kinds of logistic regression models in terms of their ability to identify and estimate covariate effects on VE. Specifically, we compare (1) the “typical” approach, which evaluates the effects of baseline covariates and *vaccination status* on clinical outcome (disease status), to (2) the proposed approach (referred to as “CoP-based”) which assesses the effects of baseline covariates and *CoP* on clinical outcome. Both approaches enable the estimation of RR (vaccinated versus control subjects) and VE in subgroups of interest.

This paper is organized as follows. Sect. "Methods" describes the modeling assumptions and the typical and CoP-based logistic regression approaches used in our analysis. Sect. "Simulation Study" evaluates the properties of these approaches (their relative ability to identify baseline covariates that impact VE) using many (5000) simulated vaccine clinical trials. Sect. "Example Analysis of a Single Hypothetical Vaccine Clinical Trial Dataset" illustrates their application to one kind of typical vaccine clinical trial (using simulated data from one representative trial). Sects. "Discussion" and "Conclusions" summarize key findings and (respectively) the implications for efficacy-based decisions.

## Methods

### Data collection and assumptions

Because they are often collected in, e.g., a randomized controlled phase 3 vaccine clinical trial, assume that the following data are available for each subject: disease status (diseased or non-diseased), vaccination status (vaccinated or control), immunogenicity biomarker value (assumed to be a CoP), and a set of baseline covariates. Disease status is a binary variable, with value 1 in diseased and 0 in non-diseased subjects, an indicator of clinical outcome set to 1 if the disease is diagnosed (by formal trial endpoint criteria) at any time during the fixed duration of the trial’s observation period. Vaccination status is a binary variable indicating treatment, with value 1 in vaccinated and 0 in control (assumed here to be placebo) subjects. The immunogenicity biomarker is a continuous variable, typically lognormally distributed (within properly defined subgroups) and typically increased by an efficacious prophylactic treatment (vaccination). Baseline covariates can be binary, categorical (ordinal or without ordering), or continuous; they are determined at baseline, prior to randomization (and vaccination).

Let ${T}_{i}^{\text{vaccinated}}$, ${T}_{j}^{\text{control}}$ be the log immunogenicity biomarker measurement (also referred to as log-titer, since neutralizing antibody titer is often used) for $i$-th vaccinated subject ($i=1,\dots , N$) and $j$-th control subject ($j=1,\dots , M$), respectively. $N$ and $M$ are the number of subjects in the vaccinated and control groups, respectively. Let ${VS}_{i}^{\text{vaccinated}}$, ${VS}_{j}^{\text{control}}$ be vaccination status, and ${DS}_{i}^{\text{vaccinated}}$, ${DS}_{j}^{\text{control}}$ be disease status for $i$-th vaccinated subject and $j$-th control subject. Let ${C}_{i,k}^{\text{vaccinated}}$, ${C}_{j,k}^{\text{control}}$ be the covariate value for $i$-th vaccinated subject, $j$-th control subject and $k$-th baseline covariate variable ($k=1,\dots , K$). $K$ represents the total number of collected baseline covariates. For a given set of $L$ independent variables ${x}_{1}, {x}_{2}, \dots , {x}_{L}$, the log-odds of disease ($y$) can be estimated by logistic regression, using a linear predictor ($lp$) as:

1 $$y=\mathrm{ log}\frac{p}{1-p}={\beta }_{0}+{\beta }_{1}{x}_{1}+{\beta }_{2}{x}_{2}+\dots +{\beta }_{L}{x}_{L}=lp,$$

where $$lp={\beta }_{0}+\sum_{l=1}^{L}{{\beta }_{l}x}_{l}$$.

Alternatively, a logistic model involving an interaction term (denoted ${\beta }_{\mathrm{1,2}}$) between independent variables ${x}_{1}$ and ${x}_{2}$ may be described as:

2 $$y=lp+{\beta }_{\mathrm{1,2}}{x}_{1}{x}_{2}.$$

The probability of disease (PoD), $p$, is

3 $$p=\frac{1}{1+{e}^{-y}}.$$

If one of the independent variables in the logistic model is log-titer, the probability of disease will be referred to as $PoD(T)$ or a PoD curve, a function of log-titer (as well as, potentially, other independent variables).

Both approaches described below, i.e., typical (not involving log-titer) and CoP-based (involving log-titer), can be used to evaluate statistical and clinical significance of covariate effects on clinical outcome as follows:

- To assess *statistical significance* of covariate effects, the test for the presence of an effect is deemed positive if either the covariate or interaction effect is proved significant (e.g., at statistical significance level $\alpha =0.05$).
- *Clinical significance* of any covariate effect depends on the application; it might be ascertained by comparing the relative health impact between subpopulations, defined with respect to the covariate of interest, using the VE difference associated with subpopulations in question. Thus, to assess clinical significance of a covariate effect associated with specific subpopulations, VE is estimated and compared across covariate-defined subgroups.

The models below are assumed to include all potentially clinically meaningful covariates, following the concept of a full covariate modeling approach [14].

### Typical approach

Independent variables used to predict disease status in the typical approach are vaccination status and baseline covariate(s) of interest. Log-odds of disease is given by Eqs. 1 and 2, with ${x}_{1}=VS$, and ${x}_{2},{x}_{3},\dots ,{x}_{L}={C}_{1},{C}_{2},\dots ,{C}_{K}$.

For illustration, in Sects. "Simulation Study" and "Example Analysis of a Single Hypothetical Vaccine Clinical Trial Dataset", only one baseline covariate, ${C}_{1}$, is considered. The following models are fitted (i.e., parameters are estimated to maximize posterior likelihood for a given dataset):

a model *not* involving interaction between the independent variables (derived from Eq. 1),

4 $$y={\beta }_{0}+{\beta }_{1}VS+{\beta }_{2}{C}_{1}, \mathrm{or }$$

a model *involving interaction* between the independent variables (derived from Eq. 2),

5 $$y={\beta }_{0}+{\beta }_{1}VS+{\beta }_{2}{C}_{1}+{\beta }_{\mathrm{1,2}}{C}_{1}VS.$$

VE can be estimated for each of the models above, using RR as:

6 $$\text{VE}=1-\text{RR}=1-\frac{{p}^{\text{vaccinated}}}{{p}^{\text{control}}},$$

where ${p}^{\text{vaccinated}}$, ${p}^{\text{control}}$ are expected values for each of the two populations, expressed as:

7 $${p}^{\text{vaccinated}}=\frac{1}{N}\cdot \sum\nolimits_{i=1}^{N}\frac{1}{1+{e}^{-{y}_{i}^{\text{vaccinated}}}},$$

8 $${p}^{\text{control}}=\frac{1}{M}\cdot \sum\nolimits_{j=1}^{M}\frac{1}{1+{e}^{-{y}_{j}^{\text{control}}}}.$$

For a given set of data, the 95% confidence interval (CI) associated with estimated VE needs to account for the uncertainty regarding the ${\beta }_{0}, {\beta }_{1}, {\beta }_{2}, {\beta }_{\mathrm{1,2}}, \dots , {\beta }_{L}$ parameters and variability in the observed data. This can be done via parametric resampling of the posterior distribution for parameters and bootstrapping the observed data in the vaccinated and control groups. The bootstrap resampling of observed data is performed on subjects: each time a subject is selected, all his/her characteristics (covariate values) are used in the estimation of VE.

### CoP-based approach

**Fig. 1:**
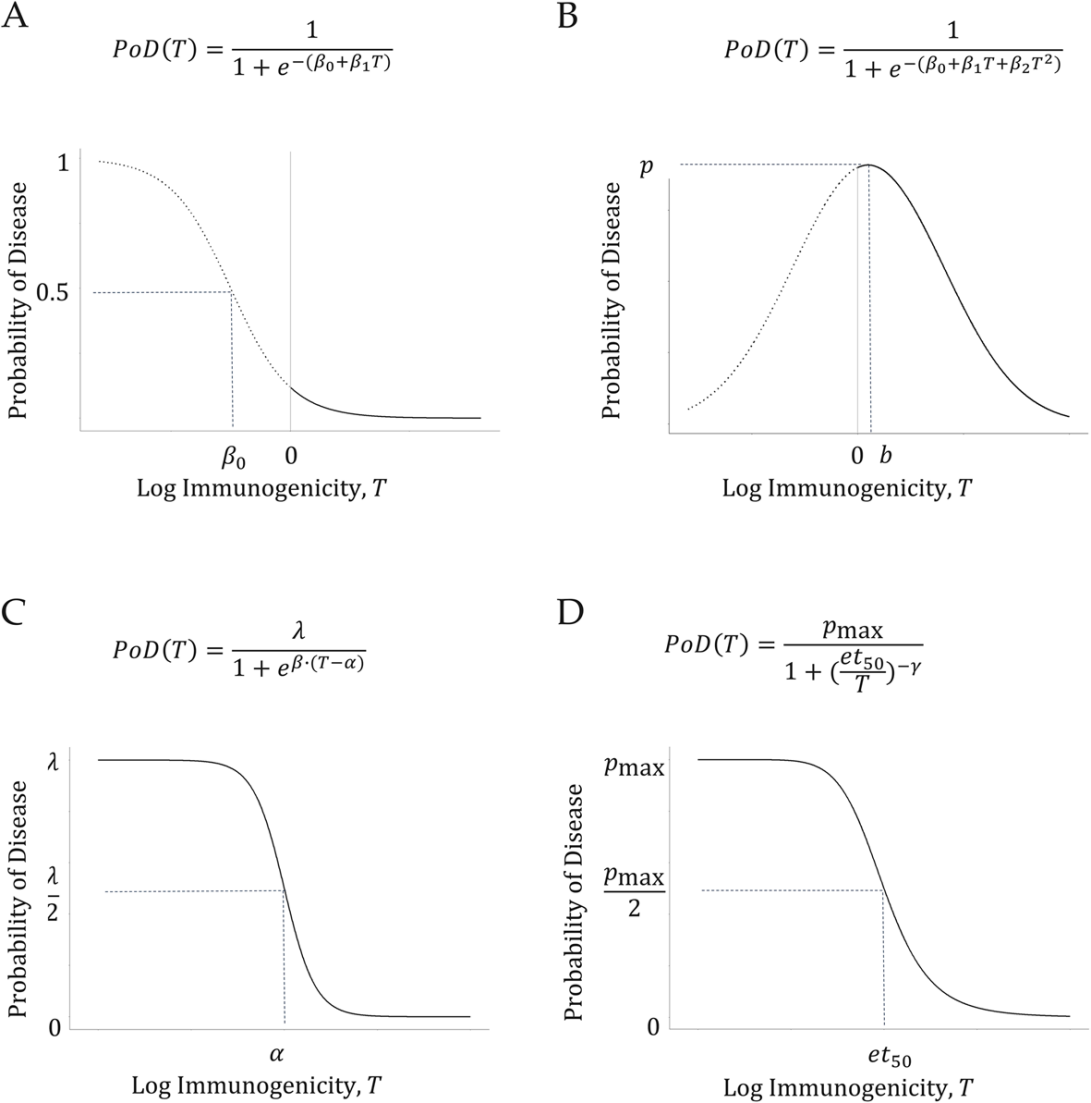
Shapes of the PoD curve (i.e., the relationship between log-titer and probability of disease). **A** logistic function with a linear term for log-titer, which is a decreasing convex function where $T>0$; **B** logistic function with a quadratic term for log-titer (with maximal probability of disease $p=\frac{1}{1+{e}^{-\left({\beta }_{0}-\frac{{{\beta }_{1}}^{2}}{4{\beta }_{2}}\right)}}$ at log-titer $b=-\frac{{\beta }_{1}}{2{\beta }_{2}}$); **C** scaled logistic function [15, 16]; **D** Hill function [12]. In infectious diseases, the biologically plausible shape of the PoD curve often corresponds to a three-parameter sigmoid function (C, D)

Several approaches have been proposed to model the relationship between the CoP and probability of disease [12, 15, 16]. In this paper, a logistic model is used for the PoD curve estimation (see Fig. 1 for comparison between logistic model and other models [12, 15, 16]).

Independent variables used to predict disease status in the CoP-based approach include (as above) the baseline covariate(s) of interest, and now also include titer (usually in the form of log-titer). Log-odds of disease is given by Eqs. 1 and 2, with ${x}_{1}=T$, and ${x}_{2},{x}_{3},\dots ,{x}_{L}={C}_{1},{C}_{2},\dots ,{C}_{K}$.

Several models can be considered, when one baseline covariate, ${C}_{1}$, is evaluated (here, again as above, for illustration):

a model with *linear* term for titer, *not* involving interaction between the independent variables,

9 $$y={\beta }_{0}+{\beta }_{1}T+{\beta }_{2}{C}_{1},$$

a model with *linear* term for titer, *involving interaction* between the independent variables,

10 $$y={\beta }_{0}+{\beta }_{1}T+{\beta }_{2}{C}_{1}+{\beta }_{\mathrm{1,2}}{C}_{1}T,$$

a model with *quadratic* term for titer, *not* involving interaction between the independent variables,

11 $$y={\beta }_{0}+{\beta }_{1}T+{\beta }_{2}{T}^{2}+{\beta }_{3}{C}_{1},{\text{or}}$$

a model with *quadratic* term for titer, *involving interaction* between the independent variables,

12 $$y={\beta }_{0}+{\beta }_{1}T+{\beta }_{2}{T}^{2}+{\beta }_{3}{C}_{1}+{\beta }_{\mathrm{2,3}}{C}_{1}{T}^{2}.$$

If the assumption of log-titer being a CoP is met (according to the Prentice framework), the effect of log-titer (linear or quadratic) is significant (among other conditions). Here, “significant” means that the coefficient involving log-titer is different from 0 at pre-specified level of statistical significance (here we adopt $\alpha =0.05$). The use of a quadratic term is here used as an illustration for a more general, non-linear relationship: in general, an unrealistically large amount of data is likely to be required to distinguish the curvature of different models in this context. The importance of logistic models with the non-linear effect of log-titer was highlighted by Callegaro and Tibaldi, 2019 [17], who demonstrated that lack of fit of a model (e.g., when using a linear effect of log-titer in the context of high VE) leads to substantial loss in power to meet Prentice criteria. Although these learnings are applicable primarily to the CoP assessment (an objective different from ours), the CoP-based approach to evaluate baseline covariate effects (proposed here) is analogous to evaluating Prentice criterion four (i.e., evaluating the effect of vaccination status when controlling for log-titer).

CoP-based VE can be determined for each of the models above using Eq. 6. The 95% CI is calculated as described above; log-titer is treated as any other covariate. Accuracy and precision of CoP-based VE, as well as the coverage probability of the respective confidence intervals, are evaluated in Sect. "Simulation Study".

A similar approach for predicting VE (without covariates) was described by Coudeville et al., 2010 [16], who used a different functional form in representing the PoD curve (Fig. 1C), and pre-vaccination and post-vaccination immune marker measurements in the vaccinated subjects (instead of immune marker data post-vaccination in the vaccinated and control groups used here).

## Simulation Study

### Overview of the simulation process

To test relative performance of the typical and the CoP-based approach in identifying impactful baseline covariates, four steps were performed:

- Step 1: Assumed true values were assigned (i) to PoD curve parameters, and (ii) to log-titer distribution parameters for all covariate-defined subgroups of the vaccinated and control group.
- Step 2: Log-titer data and baseline covariate data were generated for all vaccinated and control in silico subjects using random sampling from true distributions. Disease status was assigned to each subject randomly using the probability of disease defined by the true PoD curve.
- Step 3: To evaluate statistical significance of a baseline covariate, p-values associated with estimated regression coefficients in Eqs. 4, 5 (typical approach), and 9, 10 (CoP-based approach, with linear term for log-titer) were used as described in Sect. "Data collection and assumptions".
- Step 4: To evaluate clinical significance, the best-fitting model for each of the two approaches was selected, using the Akaike Information Criterion (AIC), from Eqs. 4 or 5 (covariate model for the typical approach), and from Eqs. 9, 10, 11, or 12 (structural and covariate model for the CoP-based approach). The selected model was used to estimate VE as described in Sects. "Typical approach" and "CoP-based approach". (By “structural model” we mean the linear, quadratic, or other form of dependence of probability of disease on log-titer.)

Steps 2 to 4 were repeated 5000 times to yield 5000 sets of data and corresponding results of covariate analysis (i.e., p-values of fitted coefficients in Step 3 and VE estimates in covariate-defined subgroups in Step 4), which were compared to the “truth” (values implied by the assigned model and parameter values).

Alternatively, the assessment of statistical significance of baseline covariate(s) may be performed using the best-fitting model, selected in Step 4. Even when numerous covariates and non-linear log-titer dependence are considered, it can be advantageous (in terms of finding the most parsimonious model) to take the stepwise-approach proposed above. This approach would be to use a linear term for log-titer in Step 3 for statistical significance assessment, and proceed to Step 4 to find the best-fitting structural model (e.g., potentially quadratic dependence on log-titer) for CoP-based VE estimation only if any covariate(s) are significant.

## Data generation and parameter estimation

**Fig. 2:**
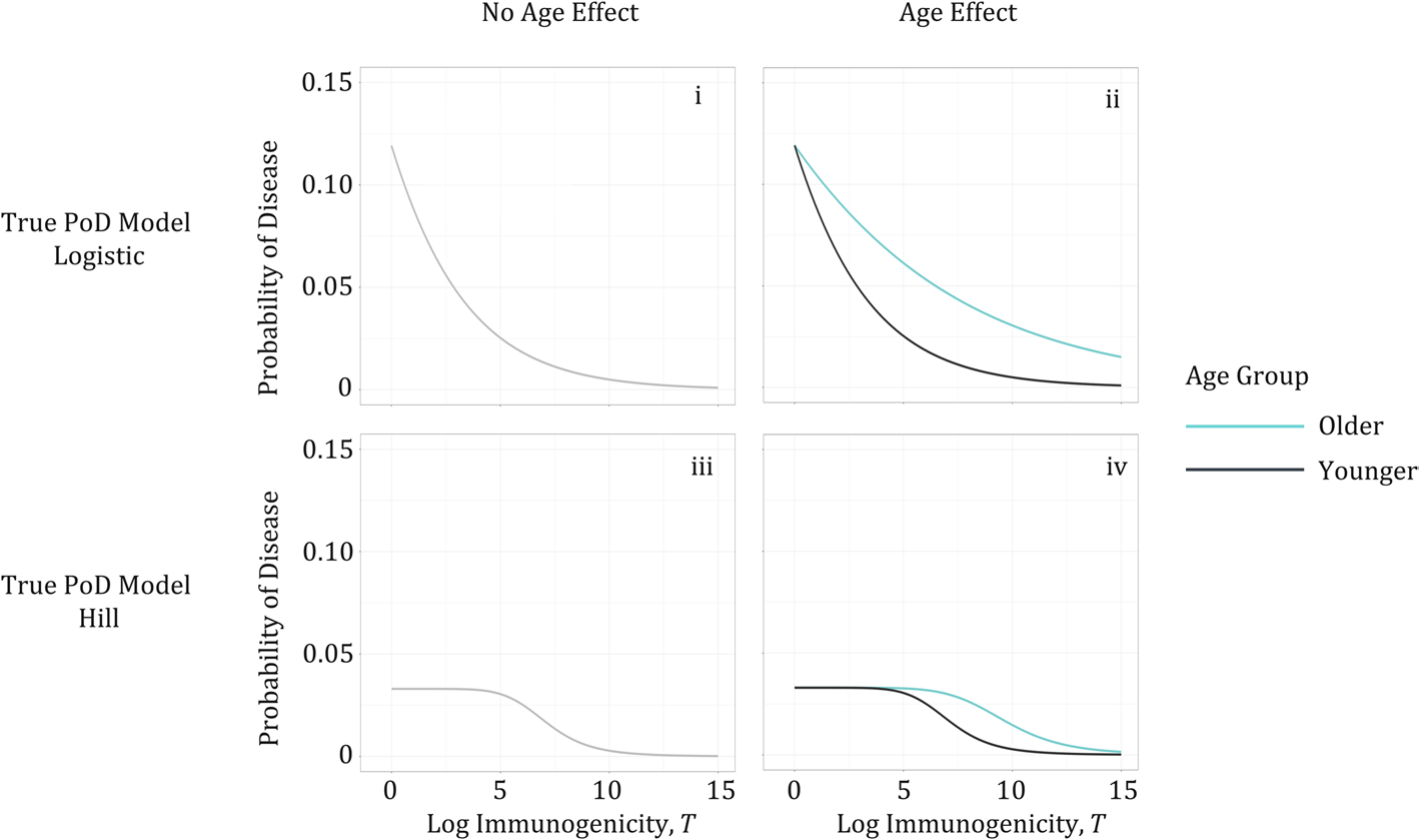
Definition of simulation scenarios i, ii, iii, iv by shapes of the true PoD curve. i: logistic function with linear term for log-titer, no age effect on the PoD curve or on VE; ii: logistic function with linear term for log-titer, age effect causes 30% difference in VE between age groups (VE of 80% in younger, 50% in older); iii: Hill function, no age effect on the PoD curve or on VE; iv: Hill function, age effect causes 30% difference in VE between age groups (VE of 80% in younger, 50% in older). The Hill function in scenarios iii and iv was chosen for simplicity as the representative example of a sigmoid function; similar results can be expected if the true PoD curve followed a scaled logistic model (Fig. 1C), given its high similarity to a Hill function [18]. Distribution of $T$ was assumed to be the same in the younger and older populations and across the scenarios, see Fig. 3

**Fig. 3:**
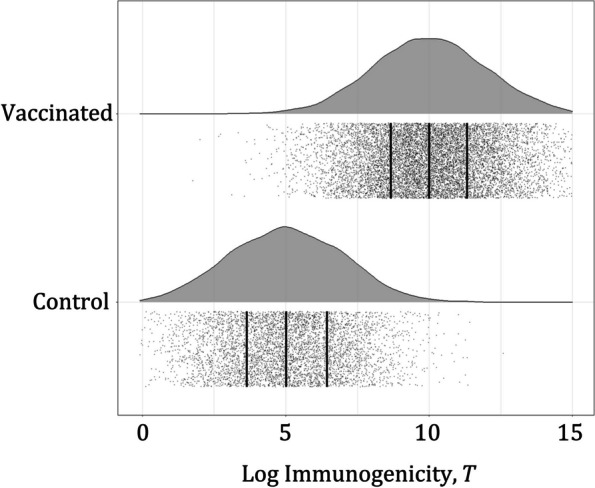
Example of immunogenicity data (log-transformed), generated in one simulated trial. Empirical probability density functions, datapoints for each subject (gray points: 10,000 vaccinated, 5000 control) and quartiles (black vertical lines). True distributions of immune marker measurements in the vaccinated and control groups used for data generation are lognormal with the same parameters in all simulated scenarios (i, ii, iii, iv) and across age groups (younger and older). The difference in VE across subgroups (80% in younger, 50% in older), in simulated scenarios ii and iv, was driven by the difference in the PoD curves

Five thousand datasets representing typical phase 3 vaccine efficacy trials were simulated for each of the four scenarios, numbered i through iv, defined by parameter values shown in Table 1, and illustrated in Figs. 1, 2 and 3. For ease of understanding and computational efficiency, only one binary baseline covariate (e.g., age group, for illustration) was considered. Difference in VE across subgroups (80% in younger, 50% in older), in simulated scenarios ii and iv, was driven by the difference in the PoD curves (Fig. 2). Simulated immunogenicity distributions of the vaccinated and control subjects were the same in younger and older subgroups in all scenarios (Fig. 3).

**Table 1:** Parameter values used as a “truth” for simulated data

**Parameter**		**Simulation scenario**			
		**i**	**ii**	**iii**	**iv**
Total number of subjects		15,000	15,000	15,000	15,000
True PoD model		Logistic	Logistic	Hill	Hill
True VE (%)	Overall	80	68	80	72
	Younger	80	80	80	80
	Older	80	50	80	50
Vaccination titers	${\text{mean}}({{{T}}}_{{{i}}}^{\text{vaccinated}})$	10	10	10	10
	${\text{sd}}({{{T}}}_{{{i}}}^{\text{vaccinated}})$	2	2	2	2
Control titers	${\text{mean}}({{{T}}}_{{{j}}}^{\text{control}})$	5	5	5	5
	${\text{sd}}({{{T}}}_{{{j}}}^{\text{control}})$	2	2	2	2
${p}_{\text{max}}$		-	-	0.033	0.033
${et}_{50}$		-	-	7.12	7.12
$\gamma$		-	-	7	7
$k$		-	-	1	1.361
${\beta }_{0}$		-2	-2	-	-
${\beta }_{1}$		-0.33	-0.33	-	-
${\beta }_{2}$		0	0	-	-
${\beta }_{\mathrm{1,2}}$		0	0.184	-	-

All scenarios: 75% of the trial population subjects are in the “younger” group and 25% in the “older” (i.e., 3:1 ratio of younger to older), with a 2:1 ratio of vaccinated to control. Parameters were chosen to obtain the indicated VE in the respective populations for each scenario while minimizing the number of parameter value differences between scenarios

Scenarios iii and iv: The Hill function was chosen for simplicity as the representative example of a sigmoid function; similar results can be expected if the true PoD curve followed a scaled logistic model (Fig. 1C), given its high similarity to a Hill function [18]

The true PoD curve was represented by a logistic function in scenarios i and ii:

13 $$PoD\left(T,A\right)=\frac{1}{1+{e}^{-\left({\beta }_{0}+{\beta }_{1}T+{\beta }_{2}A+{\beta }_{\mathrm{1,2}}A\cdot T\right)}},$$

and by a Hill function in scenarios iii and iv:

14 $$PoD\left(T,A\right)=\frac{{p}_{\text{max}}}{1+{\left(\frac{{et}_{50}{k}^{A}}{T}\right)}^{-\gamma }},$$

where $A$ represents the age group, with $A=1$ for younger participants and $A=0$ for older participants. In these forms, the ${\beta }_{\mathrm{1,2}}$ and $k$ parameters, respectively, represent a shift in log-titer required to provide a given protection for older subjects (versus that for younger).

Each simulated dataset was fitted with six logistic models (derived from Eqs. 4, 5, 9, 10, 11, 12, respectively):

15 $$p=\frac{1}{1+{e}^{-({\beta }_{0}+{\beta }_{1}VS+{\beta }_{2}A)}},$$

16 $$p=\frac{1}{1+{e}^{-({\beta }_{0}+{\beta }_{1}VS+{\beta }_{2}A+{\beta }_{\mathrm{1,2}}A\cdot VS)}},$$

17 $$p=PoD\left(T,A\right)= \frac{1}{1+{e}^{-({\beta }_{0}+{\beta }_{1}T+{\beta }_{2}A)}},$$

18 $$p=PoD\left(T,A\right)=\frac{1}{1+{e}^{-({\beta }_{0}+{\beta }_{1}T+{\beta }_{2}A+{\beta }_{\mathrm{1,2}}A\cdot T)}},$$

19 $$p=PoD\left(T,A\right)= \frac{1}{1+{e}^{-({\beta }_{0}+{\beta }_{1}T+{\beta }_{2}{T}^{2}+{\beta }_{3}A)}},$$

20 $$p=PoD\left(T,A\right)=\frac{1}{1+{e}^{-({\beta }_{0}+{\beta }_{1}T+{\beta }_{2}{T}^{2}+{\beta }_{3}A+{\beta }_{\mathrm{2,3}}A\cdot {T}^{2})}}.$$

### Assessment of age group effect

Age group, indicated by marker $A$ (with values of ${A}_{i}^{\text{vaccinated}}$, ${A}_{j}^{\text{control}}$ for $i$-th vaccinated subject, and $j$-th control subject), was termed a significant covariate in the typical approach if p-value associated with ${\beta }_{2}$ (being different from 0) in Eq. 15 or ${\beta }_{\mathrm{1,2}}$ (similarly) in Eq. 16 was less than $0.05$. In the CoP-based approach, the effect of age group was deemed significant if p-value associated with ${\beta }_{2}$ in Eq. 17 or ${\beta }_{\mathrm{1,2}}$ in Eq. 18 was less than $0.05$. (Issues of p-value correction for multiple testing are not addressed here.)

Table 2 summarizes true positive rates (percentage of simulated trials in which the effect of age group was correctly identified as significant in scenarios ii, iv), true negative rates (percentage of simulated trials in which the effect of age group was correctly identified as not significant in scenarios i, iii), positive predictive values (probability that age group impacts the outcome, when its effect was found significant), negative predictive values (probability that age group does not impact the outcome, when its effect was not found significant), and areas under the ROC curve (AUC).

**Table 2:** Results of age group effect assessment in 5000 clinical trial simulations

Metric	Logistic regression approach	Simulation scenario			
		**i**	**ii**	**iii**	**iv**
**True positive (%)**	Typical	-	100	-	99.30
	CoP-based	-	100	-	99.62
**True negative (%)**	Typical	90.62	-	91.12	-
	CoP-based	90.66	-	94.08	-
**Positive predictive value (%)**	Typical	91.42		91.79	
	CoP-based	91.46		94.39	
**Negative predictive value (%)**	Typical	100		99.24	
	CoP-based	100		99.60	

Areas under the ROC curves (AUC) are > 99.6% for all simulation scenarios

The typical approach provided true positive rates, false positive rates, negative predictive values, and AUCs that appear to be very similar to those from the CoP-based approach. When the true PoD curve was a Hill function, inclusion of the CoP predictor in the logistic regression increased positive predictive value of covariate effect detection by 2.6% from 91.8% to 94.4% (5000 simulated trials with 15,000 subjects, ~ 200 disease cases, Table 2), an effect size with potentially substantial impact (*cf.* Sect. "Discussion").

The CoP-based logistic regression with linear term for log-titer showed (Table 2) good performance of covariate effects assessment based on the p-value, even when the true relationship was a Hill function (i.e., when log-odds is a non-linear function of log-titer). Thus, for the scenarios investigated here, the model selection step (to determine the best fitting structural model, as done in Sect. "Accuracy and precision of VE estimation") is not necessary for detection of covariate effects using the criterion of statistical significance.

### Accuracy and precision of VE estimation

**Fig. 4:**
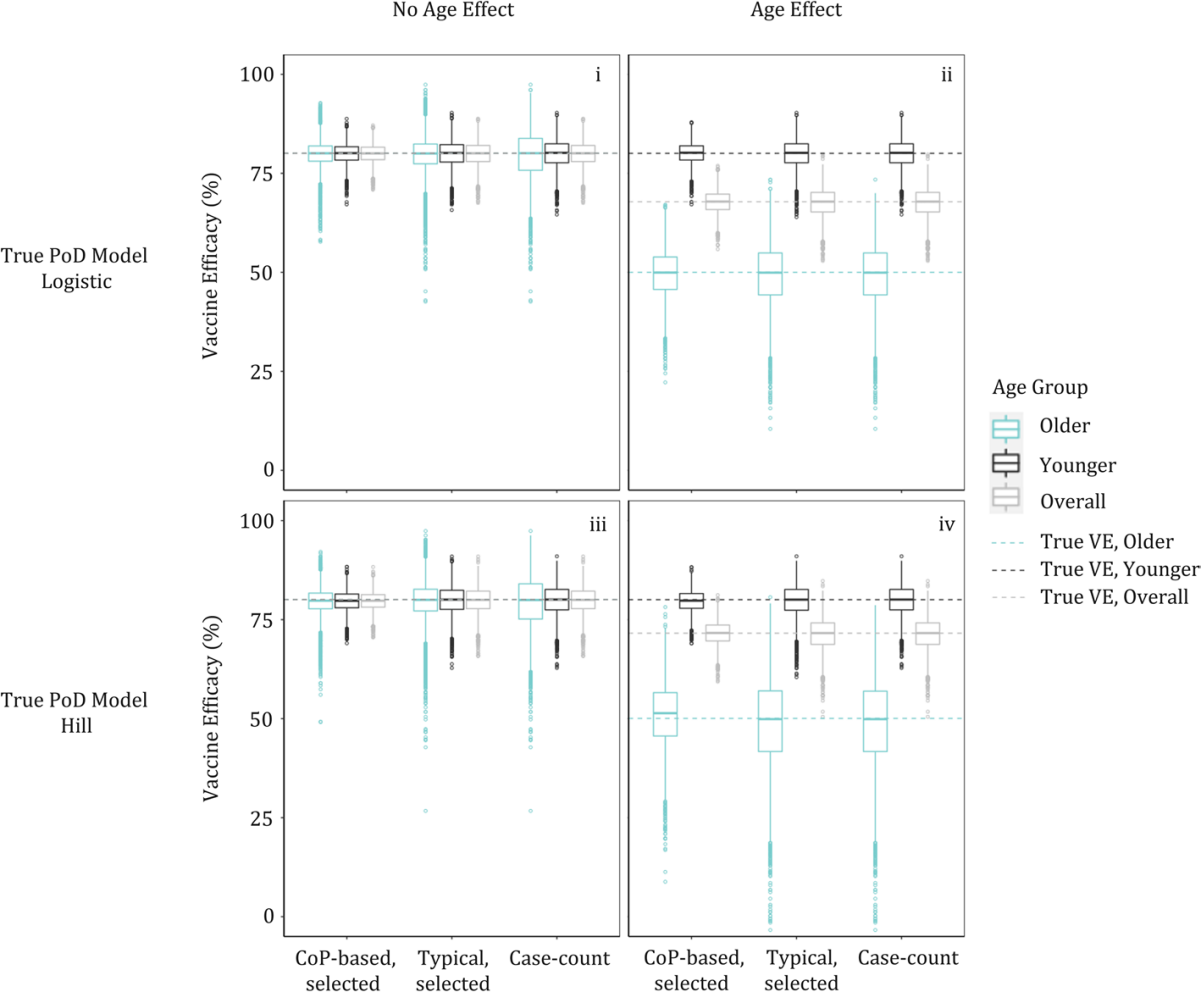
All methods are accurate; CoP-based logistic regression is generally closer to the “truth”. Age-group-specific distributions of VE point estimates for each simulated scenario using CoP-based logistic regression (for each simulated trial the best fitting model of Eqs. 17, 18, 19, 20 was selected for VE estimation, Table 3), typical logistic regression (for each simulated trial the best fitting model of Eqs. 15, 16 was selected for VE calculation, Table 3), and case-counting. The CoP-based estimate is generally closest to the “truth”: the lowest variability (narrowest interquartile range) in every scenario and subgroup is that of the CoP-based approach and the CoP-based estimate is unbiased. The term “unbiased” is used because the median is within 2% of the true value in every scenario, so any difference is unlikely to be important (clinically or statistically). In scenario iv, the CoP-based estimate in older subgroup appear to be slightly biased, but the difference between the median value (51.4%) and the true (simulated) value (50.1%) is clinically insignificant. In every scenario and subgroup, the CoP-based approach provides the lowest mean squared error (Supplementary Material, Table S1)

VE and its CI were estimated using the selected best fitting model (Eq. 15 or 16 for the typical approach; Eqs. 17, 18, 19, or 20 for the CoP-based approach; Table 3) and compared to case-counting-based estimation of VE and its CI. The simulated trials for which the selected model does not match the simulated model are generally consistent between the CoP-based and the typical approach (Table 3) and can be understood in terms of data from those trials (Supplementary Material, Figure S1). Distributions of the VE point estimates for the 5000 simulation scenarios are summarized in Fig. 4.

**Table 3:** Results of model selection for vaccine efficacy estimation

**i** True PoD model: logistic function with linear term for log-titer, no age effect				**ii** True PoD model: logistic function with linear term for log-titer, age effect			
		**Number of simulated trials, (% of simulated trials)**				**Number of simulated trials, (% of simulated trials)**	
		Model without interaction	Model with interaction			Model without interaction	Model with interaction
Typical		**4197 (84%)**	803 (16%)	Typical		28 (1%)	**4972 (99%)**
		Model without interaction	Model with interaction			Model without interaction	Model with interaction
CoP-based	Linear	**3476 (70%)**	634 (13%)	CoP-based	Linear	1 (< 1%)	**4140 (83%)**
	Quadratic	705 (14%)	185 (4%)		Quadratic	1 (< 1%)	858 (17%)
**iii** True PoD model: Hill function, no age effect				**iv** True PoD model: Hill function, age effect			
		**Number of simulated trials, (% of simulated trials)**				**Number of simulated trials, (% of simulated trials)**	
		Model without interaction	Model with interaction			Model without interaction	Model with interaction
Typical		**4181 (84%)**	819 (16%)	Typical		216 (4%)	**4784 (96%)**
		Model without interaction	Model with interaction			Model without interaction	Model with interaction
CoP-based	Linear	0 (0%)	0 (0%)	CoP-based	Linear	0 (0%)	0 (0%)
	Quadratic	**4198 (84%)**	802 (16%)		Quadratic	18 (< 1%)	**4982 (> 99%)**

Counts of simulated trials when the respective models were selected as best fitting (the option closest to the simulated “truth” is in bold) and to be used for vaccine efficacy estimation. Results are listed for all simulation scenarios. The most frequently selected model (bold) is generally consistent with the true model. The percentage of times each of the methods find a trend of interaction or lack thereof (age effect or not, resp.) is consistent between the two approaches

In the CoP-based approach, the logistic PoD curve fit (based on the selected model), combined with immunogenicity data, produced accurate point estimates of VE (Fig. 4) and well-calibrated CI (details in Supplementary Material, Tables S2-S4) for all four scenarios.

**Fig. 5:**
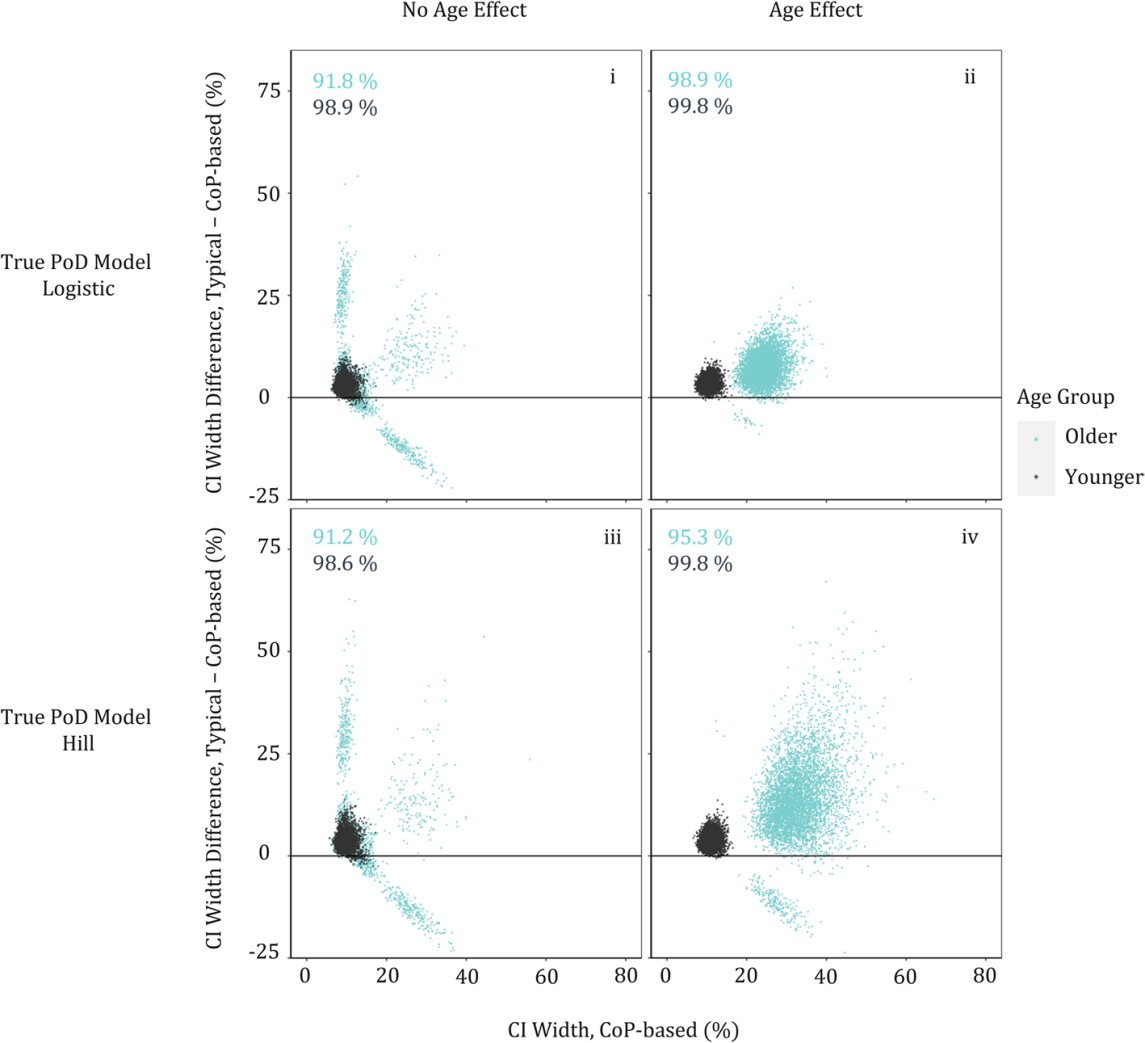
The CoP-based approach is more likely to provide better precision (narrower CI) than typical logistic regression. Comparison of widths of age-group-specific VE confidence intervals for each simulation scenario, based on the best-fit model for each simulated trial (with 2000 bootstrap samples used for CI estimation). The y-axis shows the difference between the CI width (95% upper minus lower bound) obtained by the typical approach and that obtained by the CoP-based approach; every point with difference greater than 0 is one for which the CoP-based method provides a narrower confidence interval (i.e., is more precise), and the numbers on the plots show that this happens in all four scenarios and for both subgroups (older, blue, and younger, black) over 90% of the time. The x-axis shows the CI width for the CoP-based approach. The wider spread of points (in x- and/or y- directions) is generally larger in older subjects (blue points) as there are fewer subjects in that group (25% of the population), and this is also why the CoP-based CI width is often larger (further to the right) for older subjects, especially when there is an age effect. Medians (across the 5000 simulated trials) of CoP-based CI widths for the younger group are 9.0% and 10.8% (scenarios i and iv, resp.), and, for the older group, are 9.3% and 32.2% (scenarios i and iv, resp.). Corresponding CI widths for the typical approach are 12.0% and 14.7% (younger group, scenarios i and iv, resp.), and are 12.2% and 45.1% (older group, scenarios i and iv, resp.)

In both age-defined subgroups, the VE estimation by the CoP-based approach was more precise when compared to VE estimated by the typical approach (which was more precise than case-counting in the absence of age effect, i.e., scenarios i and iii; Fig. 4 and additional details in Supplementary Material, Figure S2). The VE estimate by the CoP-based method was closer to the (simulated) “truth” than that of the typical approach for most simulated trials for these scenarios (Fig. 4). Further, Fig. 5 shows that over 90% of the time the CoP-based CI on that estimate was narrower than that obtained by the typical approach.

Implications of skipping the model selection step for accuracy and precision of VE estimation are discussed in Supplementary Material (Figures S3–S6). Even if model selection is not performed (e.g., due to computation time), *accurate* CoP-based estimates (one for each scenario i-iv) of VE are obtained (i.e., using only the Eq. 20 model to fit the simulated trials, Supplementary Material, Figure S6). However, *precision* of such estimation can be lower than that obtained using models resulting from the model selection step. If the logistic regression uses an incorrect representation of covariate effects (and, in the case of the CoP-based approach, of the structural model), then the result can be biased; if the model is not misspecified then VE predicted by typical logistic regression can be nearly identical to that obtained from case-counting.

## Example analysis of a single hypothetical vaccine clinical trial dataset

**Fig. 6:**
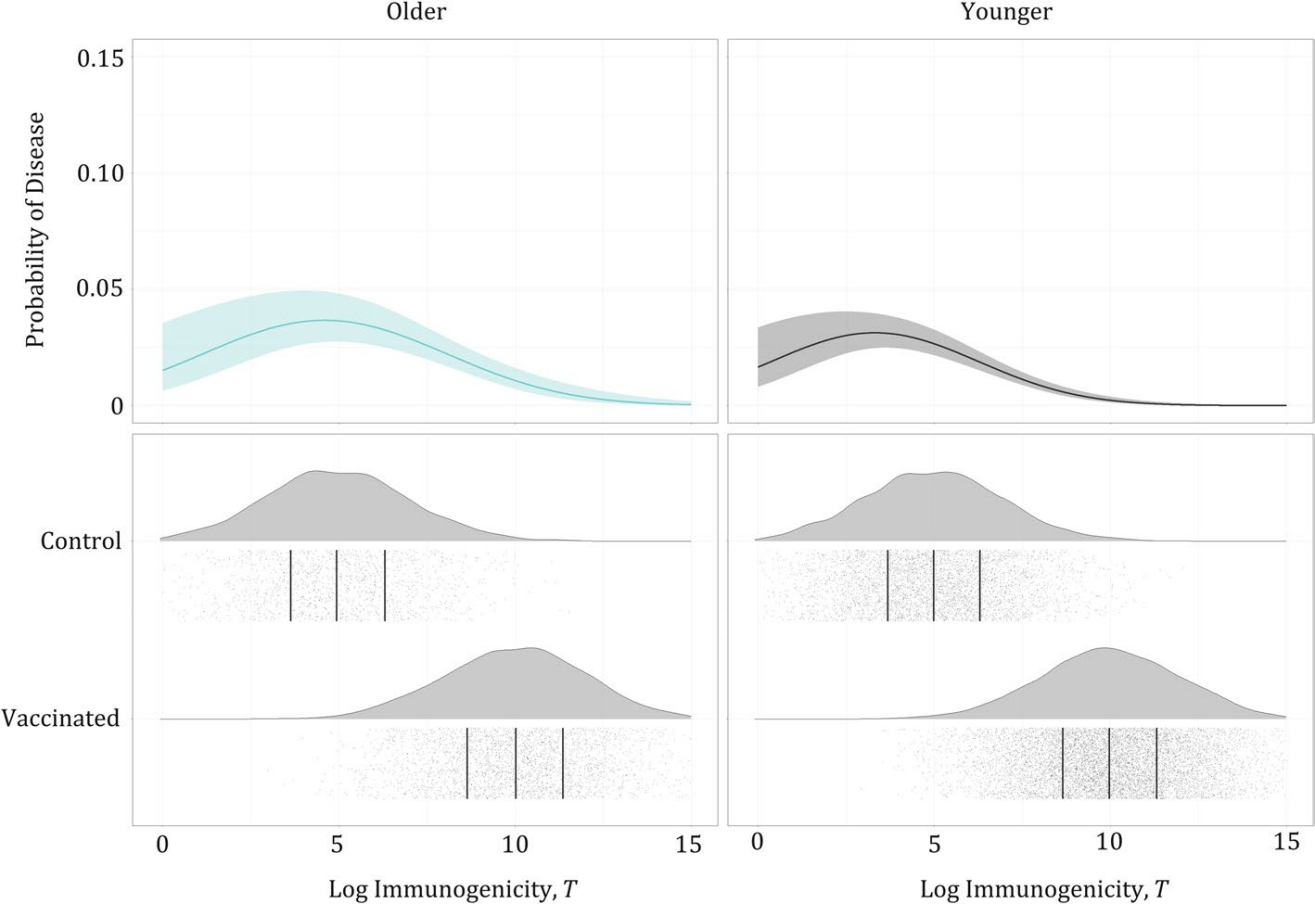
The best fitting CoP-based model shows significant effect of age group and non-linearity in titer. Estimates of the PoD curves (Eq. 20) in older and younger subjects. Even though the increasing probability of disease at lower titers is not biologically expected, the wide confidence intervals show that the model fit encompasses both flat and decreasing shapes of PoD curve (for $T<5$); it does not interfere with the ability to accurately estimate VE in each subgroup. The bottom plots show individual values of log titer by vaccination status (gray points) with respective empirical probability density functions; black vertical lines represent median, first quartile, and third quartile

To illustrate the proposed data analysis, all the methods used in the simulation study were applied to a single simulated dataset of a vaccine clinical trial (randomly selected from 5000 simulated datasets of scenario iv), which used a Hill function as the underlying true PoD curve, and the age group effect on the PoD curve leading to true VE of 50% in older and 80% in younger subjects. The simulation was of 15,000 subjects: 10,000 vaccinated, 5000 control; 3760 older, 11,240 younger; 191 diseased, 14,809 non-diseased. As shown in Fig. 6, respective immunogenicity distributions for the older and younger subjects were very similar, as there was no age effect on the simulated immunogenicity in the model used for simulation.

Table 4 shows results of age group effect assessment based on the statistical criteria. Age group was a significant predictor in all six fitted models. Table 5 reports VE estimated using the selected model for typical logistic regression (Eq. 16), and that for CoP-based logistic regression (Eq. 20), enabling assessment of clinical significance of the age group effect. CoP-based VEs were closer to true VEs and had narrower CIs than those obtained by the typical logistic regression or by case-counting. PoD curve estimates (Eq. 20) and immunogenicity data used for CoP-based VE estimation in older and younger subgroups are visualized in Fig. 6.

**Table 4:** Both logistic regression approaches (CoP-based and typical) show significant effect of age group

Model	Independent variable	Coefficient	Standard error	P-value	AIC
Typical, without interaction (Eq. 15)	(Intercept)	-4.52	0.15	< 2e-16	1970.6
	Vaccination status	1.22 (control)	0.15	6.26e-16	
	Age group	-0.59 (younger)	0.15	1.06e-04	
Typical, with interaction (Eq. 16)	(Intercept)	-4.10	0.16	< 2e-16	1953.8
	Vaccination status	0.40 (control)	0.24	0.099	
	Age group	-1.38 (younger)	0.24	7.62e-09	
	Vaccination status: age group	1.36 (control: younger)	0.32	2.16e-05	
CoP-based, linear, without interaction (Eq. 17)	(Intercept)	-2.16	0.18	< 2e-16	1916.8
	Linear log-titer	-0.25	0.02	< 2e-16	
	Age group	-0.58 (younger)	0.15	1.31e-04	
CoP-based, linear, with interaction (Eq. 18)	(Intercept)	-2.67	0.27	< 2e-16	1911.1
	Linear log-titer	-0.17	0.04	3.82e-06	
	Age group	0.22 (younger)	0.33	0.508	
	Linear log-titer: age group	-0.13 (younger)	0.05	0.005	
CoP-based, quadratic, without interaction (Eq. 19)	(Intercept)	-3.60	0.38	< 2e-16	1888.1
	Linear log-titer	0.34	0.13	0.007	
	Quadratic log-titer	-0.05	0.01	1.58e-06	
	Age group	-0.59 (younger)	0.15	9.75e-05	
CoP-based, quadratic, with interaction (Eq. 20)	(Intercept)	-4.18	0.44	< 2e-16	1878.8
	Linear log-titer	0.39	0.13	0.002	
	Quadratic log-titer	-0.04	0.01	1.50e-05	
	Age group	0.09 (younger)	0.26	0.733	
	Quadratic log-titer: age group	-0.02 (younger)	0.01	0.001	

Eqs. 16 and 20 were appropriately selected for vaccine efficacy estimation.

Age group effect assessment using statistical significance: All models with linear log-titer (Eqs. 15, 16, 17, 18) show significant effect of age group (p-values of the age group coefficient and/or of the interaction term between linear log-titer and age group are less than $0.05$).

Model selection (from the six considered) for VE estimation: The model with interaction was selected for the typical approach (Eq. 16; AIC = 1953.8) and the model with quadratic term for log-titer and with interaction was selected for the CoP-based approach (Eq. 20; AIC = 1878.8).

**Table 5:** CoP-based logistic regression is more precise than case-counting and typical logistic regression in estimating VE

Age group	Control				Vaccinated				Vaccine Efficacy, % (95% CI)		
									Outcome-based estimation		Immunogenicity-based estimation
	Subjects	Cases	Immunogenicity		Subjects	Cases	Immunogenicity		Case-count	Logistic typical (Eq. 16)	Logistic CoP-based (Eq. 20)
			GMT (95% CI)	N			GMT (95% CI)	N			
Younger	3757	89	31 (30 to 33)	3757	7483	31	1024 (992 to 1057)	7483	83 (74 to 88)	83 (74 to 88)	81 (75 to 86)
Older	1243	30	31 (28 to 33)	1243	2517	41	1031 (976 to 1090)	2517	33 (-8 to 58)	33 (-5 to 58)	60 (44 to 70)

Estimates of vaccine efficacy in older and younger subjects (with 2000 bootstrap samples used for CI estimation). True VE is 80% in younger and 50% in older subjects. The immunogenicity-based estimate allows (in general, and in this example) the more appropriate assessment of the impact of age group on vaccine efficacy.

Even though both methods correctly identify age group as a significant factor affecting VE, the CoP-based approach correctly indicates that the vaccine is efficacious in the older group, whereas the typical approach incorrectly suggests potentially negative vaccine efficacy (Tables 4 and 5). Thus, use of the typical approach could result in health authority hesitancy to license or recommend the use of this vaccine in older subjects or in a requirement for additional clinical evidence (e.g., results of an additional phase 3 study).

In contrast, if the CoP-based method is applied, a significant vaccine-induced protection in older subjects, 60% (95% CI, 44% to 70%), is estimated (appropriately).

## Discussion

Logistic regression can be reliably used to detect the effect of a binary covariate on VE. In the simulated trials of 15,000 subjects with a Hill curve as the true PoD (~ 200 disease cases), inclusion of the CoP predictor in the logistic regression increased positive predictive value of covariate effect detection by 2.6%, compared to the typical approach (PPV, i.e., probability that the true model involves the covariate when our test indicates the covariate should be included: 94.4% versus 91.8%). Thus, with a Hill curve as the true PoD, the typical approach was 46% more likely (than CoP-based) to *falsely* indicate that there is a difference in subgroups (100% minus PPV, i.e., probability of the positive test to be a false positive is 8.2% for the typical approach versus 5.6% for the CoP-based method). In other words, when the test for a covariate effect (based on statistical significance) was positive, it was more likely to be correct when using the CoP-based approach. The difference in this performance was still present (although smaller) when the true PoD curve was logistic (this was tested using a linear term for log-titer).

And, even when the typical approach detects the presence of a covariate effect, the ability of the CoP-based approach to reduce the width of VE confidence interval and to detect clinically significant effects represents a strong advantage in understanding the degree to which baseline covariates impact VE. Simulation results show that using (case-counting or) the typical logistic regression approach in the presence of a covariate effect could result in VE being underestimated enough to stop development of an efficacious vaccine. Simulations also showed that vaccine efficacy in covariate-defined subgroups was estimated accurately and more precisely if CoP data were used in the logistic regression. Phase 3 studies to evaluate vaccine efficacy are typically powered for overall case-count VE as a primary endpoint, and our analysis shows that the resulting confidence intervals of case-count VE in covariate-defined subgroups are wider than those from CoP-based methods, potentially too wide to demonstrate that VE in subgroups is significantly different from zero. Even when the covariate effect is detected, the wider CIs can result in standard methods failing to identify clinically (and even statistically) significant VE differences between subgroups (e.g., due to overlapping CIs in subgroups of interest). The loss in information (leading to the loss in precision) of the standard methods compared to the CoP-based methods is due to not incorporating the biomarker data (when a predictive biomarker exists): even use of dichotomized ("absolute CoP”) information would be likely to improve predictions (i.e., it is not just the use of a continuous versus binary predictor).

The work presented assumes immunogenicity is measured in all trial participants. In the frequent case that only a subset of non-diseased subjects is assayed for immunogenicity values, weighted logistic regression [19, 20] accounting for the case-cohort design can be used. Further research should examine how the case-cohort trial design interacts with approaches described here.

The proposed method further assumes immunogenicity data are correlated with protection, meeting Prentice criteria [7], and fully mediating the vaccine effect. In case of lack of full mediation of vaccine-induced protection through the immune response biomarker, the CoP-based approach can still be applied, and vaccination status should be included in the CoP-based logistic model (vaccination status can be added as a predictor, in addition to immune biomarker and covariate data) to account for the residual effect of the vaccine. To the extent that the biomarker is sufficiently predictive in populations of interest (despite not being fully mediating), the conclusions of work presented here could be expected to hold even without adding vaccination status as an additional predictor. While it may be reasonable in some cases to expect many of the conclusions of the work presented here to hold even with that additional predictor, future work should evaluate the implications of this modification on accuracy and precision of VE estimation and on covariate effect assessment.

## Conclusions

Inclusion of CoP data in logistic regression models provides a new method to identify baseline covariates affecting VE, offering a way to determine, sensitively and specifically, the impact of demographic, clinical, and other subject-specific characteristics on protective efficacy of a vaccine. This approach has potential to increase the precision of efficacy estimation, thus enabling increased precision and/or power in clinical trials, with concomitant enhancement of the decisions they inform.

### Supplementary Information

**Supplementary Material 1. **

## Data Availability

The datasets and the code supporting the conclusions of this article are available in the GitHub repository, https://github.com/MSDLLCpapers/simvaxpmx. A package implementing the presented methods is available in the Comprehensive R Archive Network (CRAN) repository, https://cran.r-project.org/web/packages/vaxpmx/index.html.

## Abbreviations

AIC: Akaike Information Criterion
AUC: Area under the ROC curve
CI: Confidence interval
CoP: Correlate of protection
CoR: Correlate of risk
PoD: Probability of disease
PPV: Positive predictive value
ROC: Receiver operating characteristic
RR: Relative risk
VE: Vaccine efficacy
