# Supplementary material for: Elucidating vaccine efficacy using a correlate of protection, demographics, and logistic regression
Source: BMC Med Res Methodol. 2024 Apr 30;24:101. doi: 10.1186/s12874-024-02197-3 (PMC11059665; doi:10.1186/s12874-024-02197-3)
Supplement: Supplementary file 1 — Supplementary Material 1. [file 12874_2024_2197_MOESM1_ESM.docx]

**Table S1:** **Mean squared error (MSE) of VE (%) for best-fitting models in each simulation scenario.**

| \| i True PoD model: logistic function with linear term for log-titer, no age effect \| \| \| \|  \| \| --- \| --- \| --- \| --- \| --- \| \|  \|  \| **MSE** \| \|  \| \|  \|  \| Younger \| Older \| Overall \| \| CoP-based \|  \| 6.5 \| 16.1 \| 5.4 \| \| Typical \|  \| 11.2 \| 28.7 \| 9.4 \| \| Case-counting \|  \| 12.7 \| 40.6 \| 9.4 \| | \| ii True PoD model: logistic function with linear term for log-titer, age effect \| \| \| \|  \| \| --- \| --- \| --- \| --- \| --- \| \|  \|  \| **MSE** \| \|  \| \|  \|  \| Younger \| Older \| Overall \| \| CoP-based \|  \| 7.3 \| 36.9 \| 8.4 \| \| Typical \|  \| 13.1 \| 65.6 \| 13.8 \| \| Case-counting \|  \| 12.7 \| 64.9 \| 13.8 \| |
| --- | --- | --- | --- | --- | --- | --- | --- | --- | --- | --- | --- | --- | --- | --- | --- | --- | --- | --- | --- | --- | --- | --- | --- | --- | --- | --- | --- | --- | --- | --- | --- | --- | --- | --- | --- | --- | --- | --- | --- | --- | --- | --- | --- | --- | --- | --- | --- | --- | --- | --- | --- | --- | --- | --- | --- | --- | --- | --- | --- | --- | --- |
|  |  |
| \| iii True PoD model: Hill function, no age effect \| \| \| \|  \| \| --- \| --- \| --- \| --- \| --- \| \|  \|  \| **MSE** \| \|  \| \|  \|  \| Younger \| Older \| Overall \| \| CoP-based \|  \| 7.0 \| 17.1 \| 5.8 \| \| Typical \|  \| 13.1 \| 33.9 \| 10.9 \| \| Case-counting \|  \| 14.7 \| 48.8 \| 10.9 \| | \| iv True PoD model: Hill function, age effect \| \| \| \|  \| \| --- \| --- \| --- \| --- \| --- \| \|  \|  \| **MSE** \| \|  \| \|  \|  \| Younger \| Older \| Overall \| \| CoP-based \|  \| 7.8 \| 68.9 \| 8.8 \| \| Typical \|  \| 16.6 \| 154.8 \| 16.5 \| \| Case-counting \|  \| 14.7 \| 145.6 \| 16.5 \| |

MSEs are calculated using age-group-specific distributions of VE point estimates for (1) CoP-based logistic regression (for each simulated trial the best fitting model of Eqs. 17, 18, 19, 20 for that particular trial was selected for VE estimation, Table 3), (2) typical logistic regression (for each simulated trial the best fitting model of Eqs. 15, 16 for that particular trial was selected for VE calculation, Table 3), and (3) case-counting. Results are listed for all scenarios, with true PoD model as i: logistic function with linear term for log-titer, no age effect; ii: logistic function with linear term for log-titer, age effect; iii: Hill function, no age effect; iv: Hill function, age effect.

**Table S2: Coverage probability of overall VE confidence intervals for all six fitted models in each simulation scenario.**

| \| i  True PoD model: logistic function with linear term for log-titer, no age effect \| \| \| \| \| --- \| --- \| --- \| --- \| \|  \|  \| **Coverage probability, %** \| \| \|  \|  \|  \| \| \|  \|  \| Model without interaction \| Model with interaction \| \| Typical \|  \| **94.9** \| 95.1 \| \|  \|  \|  \|  \| \|  \|  \| Model without interaction \| Model with interaction \| \| CoP-based \| Linear \| **95.1** \| 95.2 \| \| Quadratic \| 95.0 \| 95.2 \| | \| ii  True PoD model: logistic function with linear term for log-titer, age effect \| \| \| \| \| --- \| --- \| --- \| --- \| \|  \|  \| **Coverage probability, %** \| \| \|  \|  \|  \| \| \|  \|  \| Model without interaction \| Model with interaction \| \| Typical \|  \| 94.8 \| **94.9** \| \|  \|  \|  \|  \| \|  \|  \| Model without interaction \| Model with interaction \| \| CoP-based \| Linear \| 94.2 \| **94.9** \| \| Quadratic \| 95.0 \| 95.3 \| |
| --- | --- | --- | --- | --- | --- | --- | --- | --- | --- | --- | --- | --- | --- | --- | --- | --- | --- | --- | --- | --- | --- | --- | --- | --- | --- | --- | --- | --- | --- | --- | --- | --- | --- | --- | --- | --- | --- | --- | --- | --- | --- | --- | --- | --- | --- | --- | --- | --- | --- | --- | --- | --- | --- | --- | --- | --- | --- | --- | --- | --- | --- | --- | --- | --- | --- | --- | --- | --- | --- | --- | --- |
|  |  |
| \| iii True PoD model: Hill function, no age effect \| \| \| \| \| --- \| --- \| --- \| --- \| \|  \|  \| **Coverage probability, %** \| \| \|  \|  \|  \| \| \|  \|  \| Model without interaction \| Model with interaction \| \| Typical \|  \| **95.0** \| 95.4 \| \|  \|  \|  \|  \| \|  \|  \| Model without interaction \| Model with interaction \| \| CoP-based \| Linear \| 73.4 \| 72.8 \| \| Quadratic \| **94.1** \| 94.2 \| | \| iv True PoD model: Hill function, age effect \| \| \| \| \| --- \| --- \| --- \| --- \| \|  \|  \| **Coverage probability, %** \| \| \|  \|  \|  \| \| \|  \|  \| Model without interaction \| Model with interaction \| \| Typical \|  \| 95.1 \| **95.2** \| \|  \|  \|  \|  \| \|  \|  \| Model without interaction \| Model with interaction \| \| CoP-based \| Linear \| 91.5 \| 90.9 \| \| Quadratic \| 94.5 \| **94.9** \| |

Best fitting models for majority of simulated trials highlighted in bold (see Table 3). Results listed for all scenarios, with true PoD model as i: logistic function with linear term for log-titer, no age effect; ii: logistic function with linear term for log-titer, age effect; iii: Hill function, no age effect; iv: Hill function, age effect.

**Table S3:** **Coverage probability of VE in younger confidence intervals for all six fitted models in each simulation scenario.**

| \| i True PoD model: logistic function with linear term for log-titer, no age effect \| \| \| \| \| --- \| --- \| --- \| --- \| \|  \|  \| **Coverage probability, %** \| \| \|  \|  \| **Younger subjects** \| \| \|  \|  \| Model without interaction \| Model with interaction \| \| Typical \|  \| **94.9** \| 94.7 \| \|  \|  \|  \|  \| \|  \|  \| Model without interaction \| Model with interaction \| \| CoP-based \| Linear \| **94.9** \| 94.8 \| \| Quadratic \| 95.0 \| 95.2 \| | \| ii  True PoD model: logistic function with linear term for log-titer, age effect \| \| \| \| \| --- \| --- \| --- \| --- \| \|  \|  \| **Coverage probability, %** \| \| \|  \|  \| **Younger subjects** \| \| \|  \|  \| Model without interaction \| Model with interaction \| \| Typical \|  \| 2.5 \| **94.7** \| \|  \|  \|  \|  \| \|  \|  \| Model without interaction \| Model with interaction \| \| CoP-based \| Linear \| 0.26 \| **94.7** \| \| Quadratic \| 0.2 \| 94.0 \| |
| --- | --- | --- | --- | --- | --- | --- | --- | --- | --- | --- | --- | --- | --- | --- | --- | --- | --- | --- | --- | --- | --- | --- | --- | --- | --- | --- | --- | --- | --- | --- | --- | --- | --- | --- | --- | --- | --- | --- | --- | --- | --- | --- | --- | --- | --- | --- | --- | --- | --- | --- | --- | --- | --- | --- | --- | --- | --- | --- | --- | --- | --- | --- | --- | --- | --- | --- | --- | --- | --- | --- | --- |
|  |  |
| \| iii True PoD model: Hill function, no age effect \| \| \| \| \| --- \| --- \| --- \| --- \| \|  \|  \| **Coverage probability, %** \| \| \|  \|  \| **Younger subjects** \| \| \|  \|  \| Model without interaction \| Model with interaction \| \| Typical \|  \| **95.0** \| 94.7 \| \|  \|  \|  \|  \| \|  \|  \| Model without interaction \| Model with interaction \| \| CoP-based \| Linear \| 73.7 \| 80.7 \| \| Quadratic \| **94.4** \| 94.6 \| | \| iv True PoD model: Hill function, age effect \| \| \| \| \| --- \| --- \| --- \| --- \| \|  \|  \| **Coverage probability, %** \| \| \|  \|  \| **Younger subjects** \| \| \|  \|  \| Model without interaction \| Model with interaction \| \| Typical \|  \| 30.8 \| **94.7** \| \|  \|  \|  \|  \| \|  \|  \| Model without interaction \| Model with interaction \| \| CoP-based \| Linear \| 0.9 \| 80.9 \| \| Quadratic \| 7.0 \| **94.7** \| |

Best fitting models for majority of simulated trials highlighted in bold (see Table 3). Results listed for all scenarios, with true PoD model as i: logistic function with linear term for log-titer, no age effect; ii: logistic function with linear term for log-titer, age effect; iii: Hill function, no age effect; iv: Hill function, age effect.

**Table S4:** **Coverage probability of VE in older confidence intervals for all six fitted models in each simulation scenario.**

| \| i True PoD model: logistic function with linear term for log-titer, no age effect \| \| \| \| \| --- \| --- \| --- \| --- \| \|  \|  \| **Coverage probability, %** \| \| \|  \|  \| **Older subjects** \| \| \|  \|  \| Model without interaction \| Model with interaction \| \| Typical \|  \| **95.0** \| 95.1 \| \|  \|  \|  \|  \| \|  \|  \| Model without interaction \| Model with interaction \| \| CoP-based \| Linear \| **95.0** \| 94.9 \| \| Quadratic \| 95.0 \| 95.6 \| | \| ii True PoD model: logistic function with linear term for log-titer, age effect \| \| \| \| \| --- \| --- \| --- \| --- \| \|  \|  \| **Coverage probability, %** \| \| \|  \|  \| **Older subjects** \| \| \|  \|  \| Model without interaction \| Model with interaction \| \| Typical \|  \| 3.2 \| **94.8** \| \|  \|  \|  \|  \| \|  \|  \| Model without interaction \| Model with interaction \| \| CoP-based \| Linear \| 0.1 \| **95.2** \| \| Quadratic \| 0.1 \| 95.3 \| |
| --- | --- | --- | --- | --- | --- | --- | --- | --- | --- | --- | --- | --- | --- | --- | --- | --- | --- | --- | --- | --- | --- | --- | --- | --- | --- | --- | --- | --- | --- | --- | --- | --- | --- | --- | --- | --- | --- | --- | --- | --- | --- | --- | --- | --- | --- | --- | --- | --- | --- | --- | --- | --- | --- | --- | --- | --- | --- | --- | --- | --- | --- | --- | --- | --- | --- | --- | --- | --- | --- | --- | --- |
|  |  |
| \| iii True PoD model: Hill function, no age effect \| \| \| \| \| --- \| --- \| --- \| --- \| \|  \|  \| **Coverage probability, %** \| \| \|  \|  \| **Older subjects** \| \| \|  \|  \| Model without interaction \| Model with interaction \| \| Typical \|  \| **95.0** \| 95.5 \| \|  \|  \|  \|  \| \|  \|  \| Model without interaction \| Model with interaction \| \| CoP-based \| Linear \| 74.3 \| 93.4 \| \| Quadratic \| **74.3** \| 95.1 \| | \| iv True PoD model: Hill function, age effect \| \| \| \| \| --- \| --- \| --- \| --- \| \|  \|  \| **Coverage probability, %** \| \| \|  \|  \| **Older subjects** \| \| \|  \|  \| Model without interaction \| Model with interaction \| \| Typical \|  \| 1.8 \| **95.2** \| \|  \|  \|  \|  \| \|  \|  \| Model without interaction \| Model with interaction \| \| CoP-based \| Linear \| 0.2 \| 97.6 \| \| Quadratic \| 0.2 \| **95.3** \| |

Best fitting models for majority of simulated trials highlighted in bold (see Table 3). Results listed for all scenarios, with true PoD model as i: logistic function with linear term for log-titer, no age effect; ii: logistic function with linear term for log-titer, age effect; iii: Hill function, no age effect; iv: Hill function, age effect.

**Table S5:** **Mean squared error (MSE) of VE (%) for models 16 and 20 in each simulation scenario.**

| \| i True PoD model: logistic function with linear term for log-titer, no age effect \| \| \| \|  \| \| --- \| --- \| --- \| --- \| --- \| \|  \|  \| **MSE** \| \|  \| \|  \|  \| Younger \| Older \| Overall \| \| CoP-based \|  \| 7.5 \| 22.3 \| 5.6 \| \| Typical \|  \| 12.7 \| 40.6 \| 9.4 \| \| Case-counting \|  \| 12.7 \| 40.6 \| 9.4 \| | \| ii True PoD model: logistic function with linear term for log-titer, age effect \| \| \| \|  \| \| --- \| --- \| --- \| --- \| --- \| \|  \|  \| **MSE** \| \|  \| \|  \|  \| Younger \| Older \| Overall \| \| CoP-based \|  \| 8.0 \| 33.6 \| 8.3 \| \| Typical \|  \| 12.7 \| 64.9 \| 13.8 \| \| Case-counting \|  \| 12.7 \| 64.9 \| 13.8 \| |
| --- | --- | --- | --- | --- | --- | --- | --- | --- | --- | --- | --- | --- | --- | --- | --- | --- | --- | --- | --- | --- | --- | --- | --- | --- | --- | --- | --- | --- | --- | --- | --- | --- | --- | --- | --- | --- | --- | --- | --- | --- | --- | --- | --- | --- | --- | --- | --- | --- | --- | --- | --- | --- | --- | --- | --- | --- | --- | --- | --- | --- | --- |
|  |  |
| \| iii True PoD model: Hill function, no age effect \| \| \| \|  \| \| --- \| --- \| --- \| --- \| --- \| \|  \|  \| **MSE** \| \|  \| \|  \|  \| Younger \| Older \| Overall \| \| CoP-based \|  \| 7.8 \| 24.2 \| 5.8 \| \| Typical \|  \| 14.7 \| 48.8 \| 10.9 \| \| Case-counting \|  \| 14.7 \| 48.8 \| 10.9 \| | \| iv True PoD model: Hill function, age effect \| \| \| \|  \| \| --- \| --- \| --- \| --- \| --- \| \|  \|  \| **MSE** \| \|  \| \|  \|  \| Younger \| Older \| Overall \| \| CoP-based \|  \| 7.7 \| 68.3 \| 8.8 \| \| Typical \|  \| 14.7 \| 145.6 \| 16.5 \| \| Case-counting \|  \| 14.7 \| 145.6 \| 16.5 \| |

MSEs are calculated using age-group-specific distributions of VE point estimates for each simulated scenario using (1) CoP-based logistic regression (quadratic model with an interaction, Eq. 20, used for VE calculation), (2) typical logistic regression (model with an interaction, Eq. 16, used for VE calculation), and (3) case-counting. Results are listed for all scenarios, with true PoD model as i: logistic function with linear term for log-titer, no age effect; ii: logistic function with linear term for log-titer, age effect; iii: Hill function, no age effect; iv: Hill function, age effect.

**
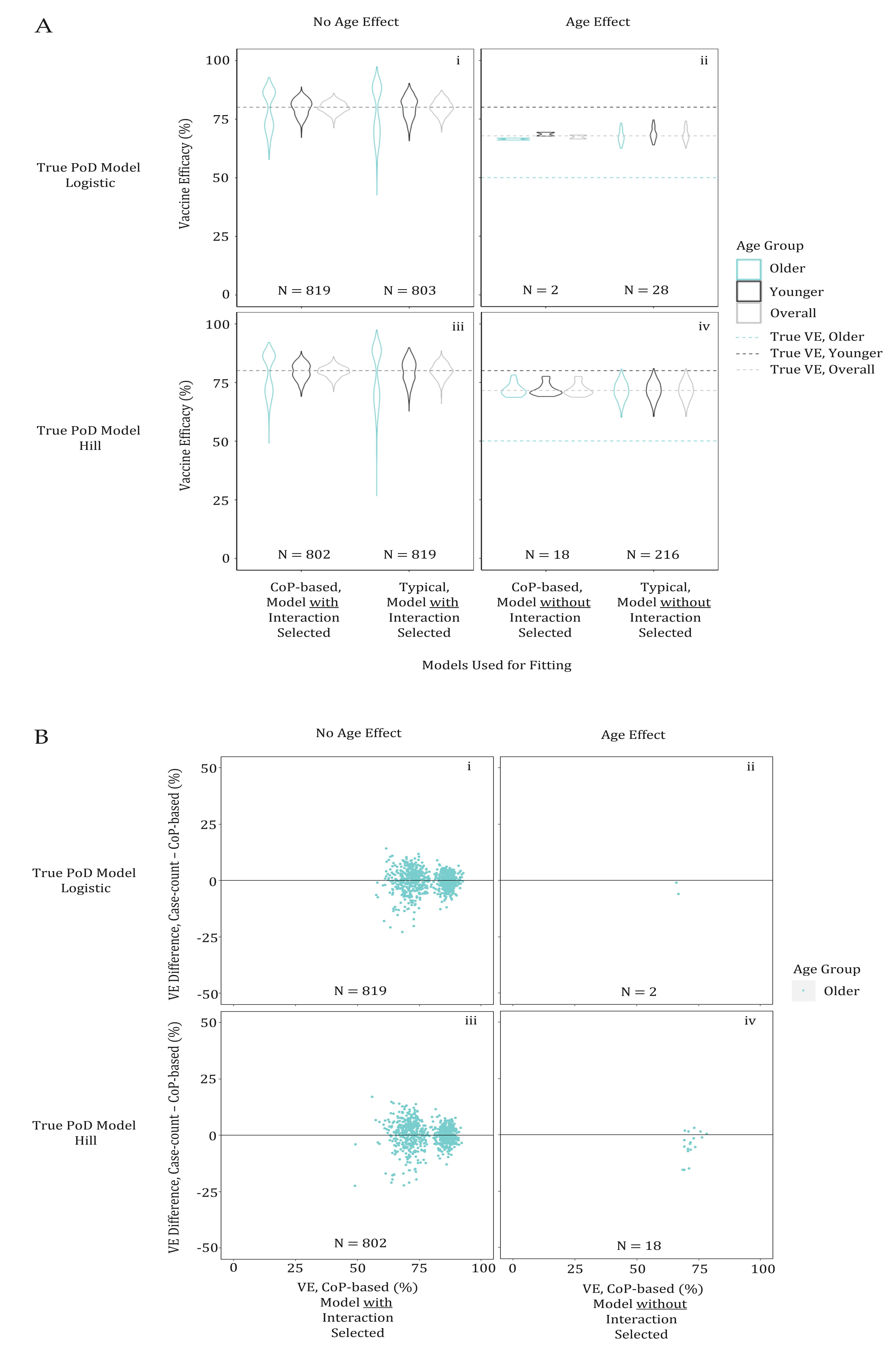
**

**Figure S1.**

A: Age group specific distributions of VE point estimates for each simulated scenario using CoP-based logistic regression (selected best-fit model used for VE calculation), and typical logistic regression (selected best-fit model used for VE calculation) for subsets of simulated trials (N, 2 to 819, out of 5000) for which the selected model is not consistent with the true model. In scenarios with no simulated age effect (i, iii), the bimodal shape of VE distributions (especially in older group, due to higher variability) leads to apparent differences in VE point estimates between younger and older groups; these differences are not always statistically significant (false positive rate is lower than 819/5000, Table 2). In scenarios with simulated age effect (ii, iv), there are no apparent differences in VE point estimates between younger and older groups in presented subsets of simulated trials, which are mostly outliers (as shown in Figure 4).

B: VE point estimates by case-counting are consistent with CoP-based VE point estimates (selected best-fit model used for VE calculation) in older subjects and for subsets of simulated trials for which the selected model is not consistent with the true model.

**
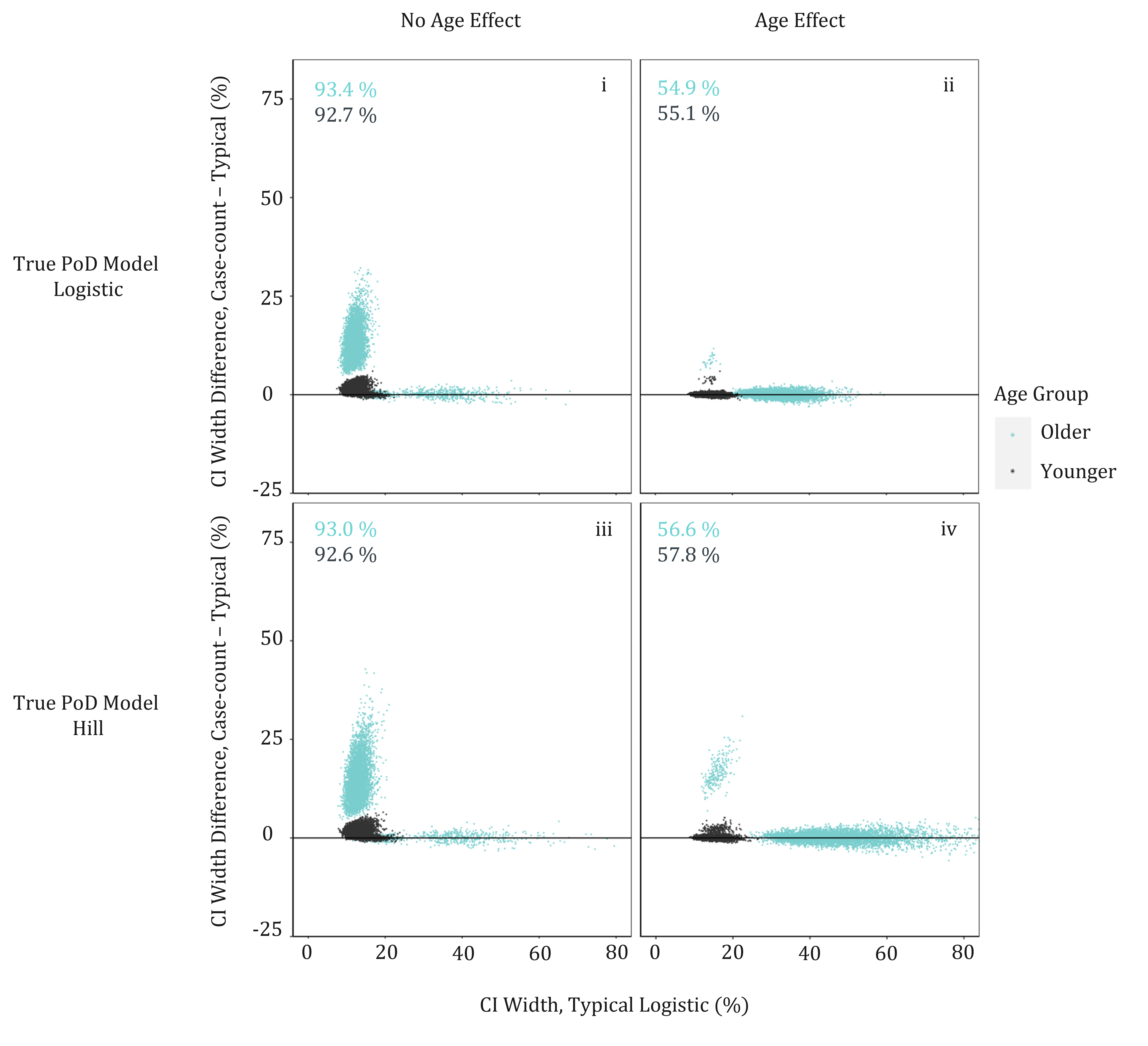
**

**Figure S2:** **Logistic-based CI (typical approach, without use of CoP data) is generally narrower than case-counting-based CI when there is no age effect. When there are differences in subgroups’ VE, precision of typical logistic regression and case-counting is similar.**

Comparison of widths of age-group-specific VE confidence intervals for each simulation scenario, based on case-counting and the typical best-fit model for each simulated trial. The y-axis shows the difference between the CI width (95% upper minus lower bound) obtained by the case-counting and that obtained by the typical logistic regression; every point with difference greater than 0 is one for which the CoP-based method provides a narrower confidence interval (i.e., is more precise), and the numbers on the plots show that this happens in scenarios with no simulated age effect and for both subgroups (older, blue, and younger, black) over 54% of the time. The x-axis shows the CI width for the typical logistic regression. Medians (across the 5000 simulated trials) of typical logistic-based CI widths for the younger group are 12.0% and 14.7% (scenarios i and iv, resp.), and, for the older group, are 12.2% and 45.1% (scenarios i and iv, resp.). Corresponding CI widths for case-counting are 13.7% and 14.9% (younger group, scenarios i and iv, resp.), and are 24.8% and 45.2% (older group, scenarios i and iv, resp.).

**
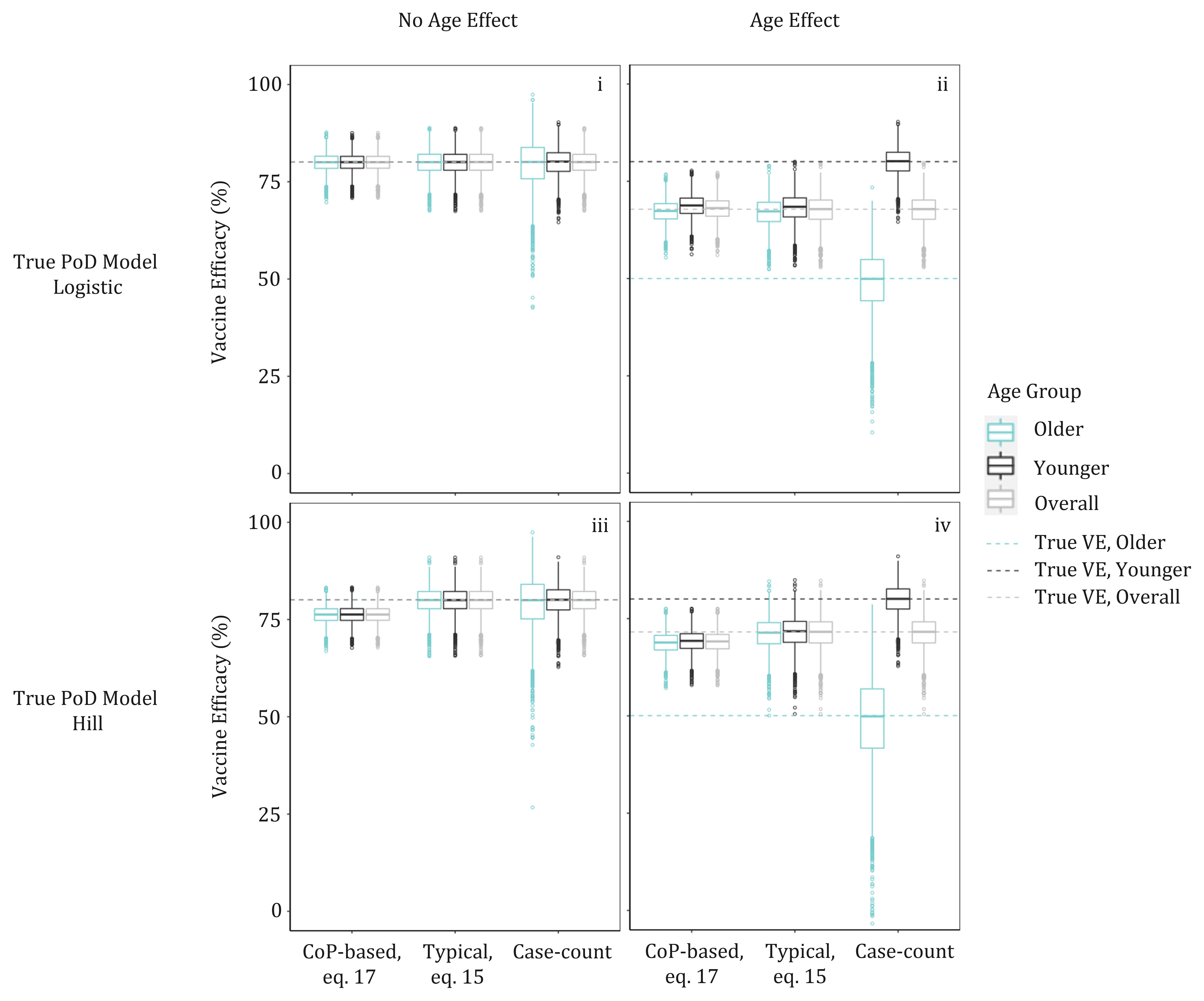
**

**Figure S3:** **Models 15 and 17 provide biased estimates of VE in some scenarios.**

Age-group-specific distributions of VE point estimates for each simulated scenario using CoP-based logistic regression (linear model without an interaction, Eq. 17, used for VE estimation), typical logistic regression (model without an interaction, Eq. 15, used for VE estimation), and case-counting. VE estimation by Eq. 17 is accurate only if true PoD model is logistic with no age effect (i); VE in subgroups is biased if VE differs across subgroups (ii, iv) or if true PoD model is Hill (iii). VE estimation by Eq. 15 is accurate in scenarios with no age effect (i, iii); VE in subgroups is biased if VE differs across subgroups (ii, iv).

**
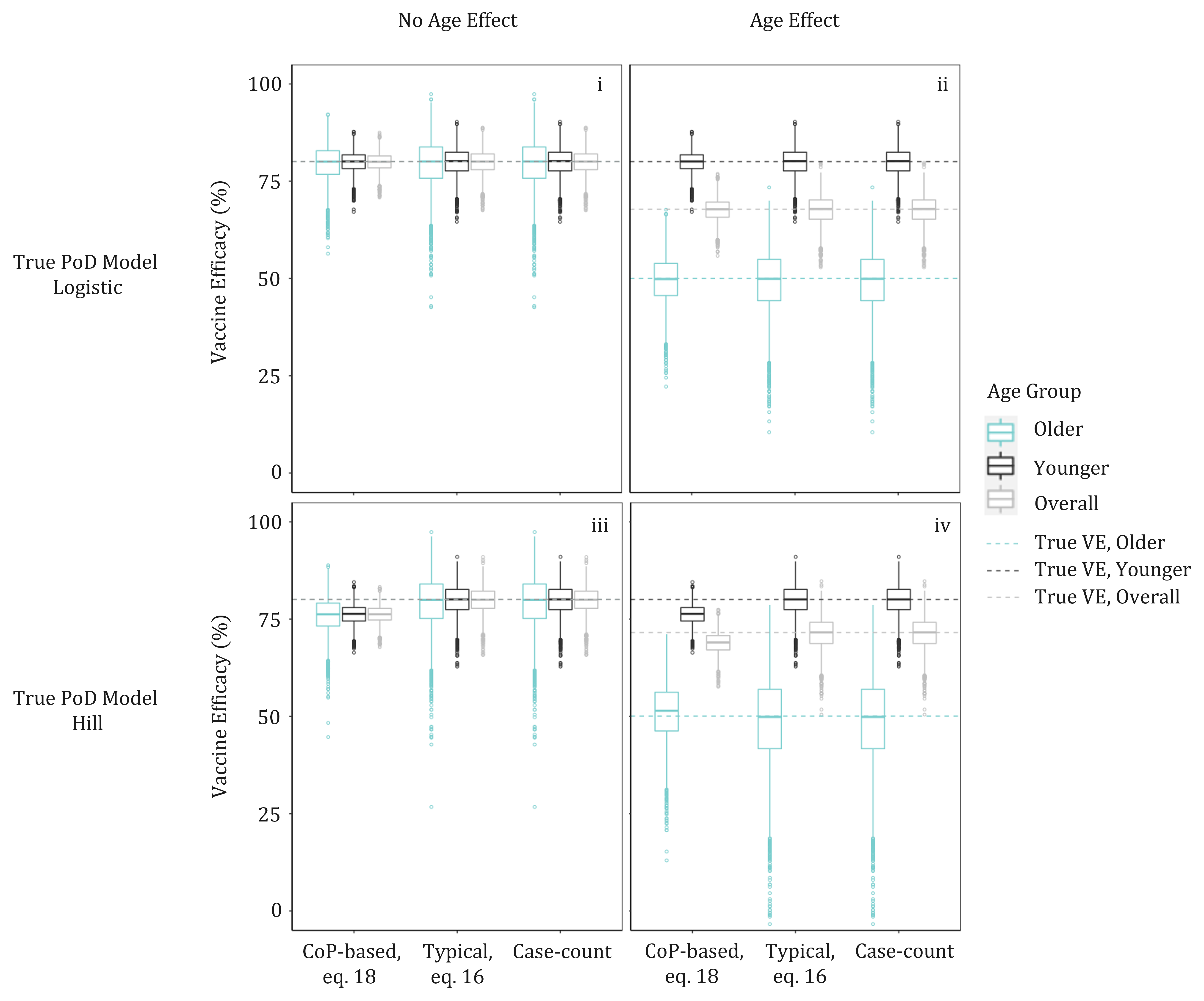
**

**Figure S4:** **Model 18 provides biased estimates of VE in some scenarios, while** **model 16 provides accurate (unbiased) estimates of VE in all scenarios.**

Age-group-specific distributions of VE point estimates for each simulated scenario using CoP-based logistic regression (linear model with an interaction, Eq. 18, used for VE estimation), typical logistic regression (model with an interaction, Eq. 16, used for VE estimation), and case-counting. VE estimation by Eq. 18 is accurate only if true PoD model is logistic (i, ii); biased if true PoD model is Hill (iii, iv). VE estimation by Eq. 16 is accurate in all scenarios. VE estimation by case-counting is accurate in all scenarios.

**
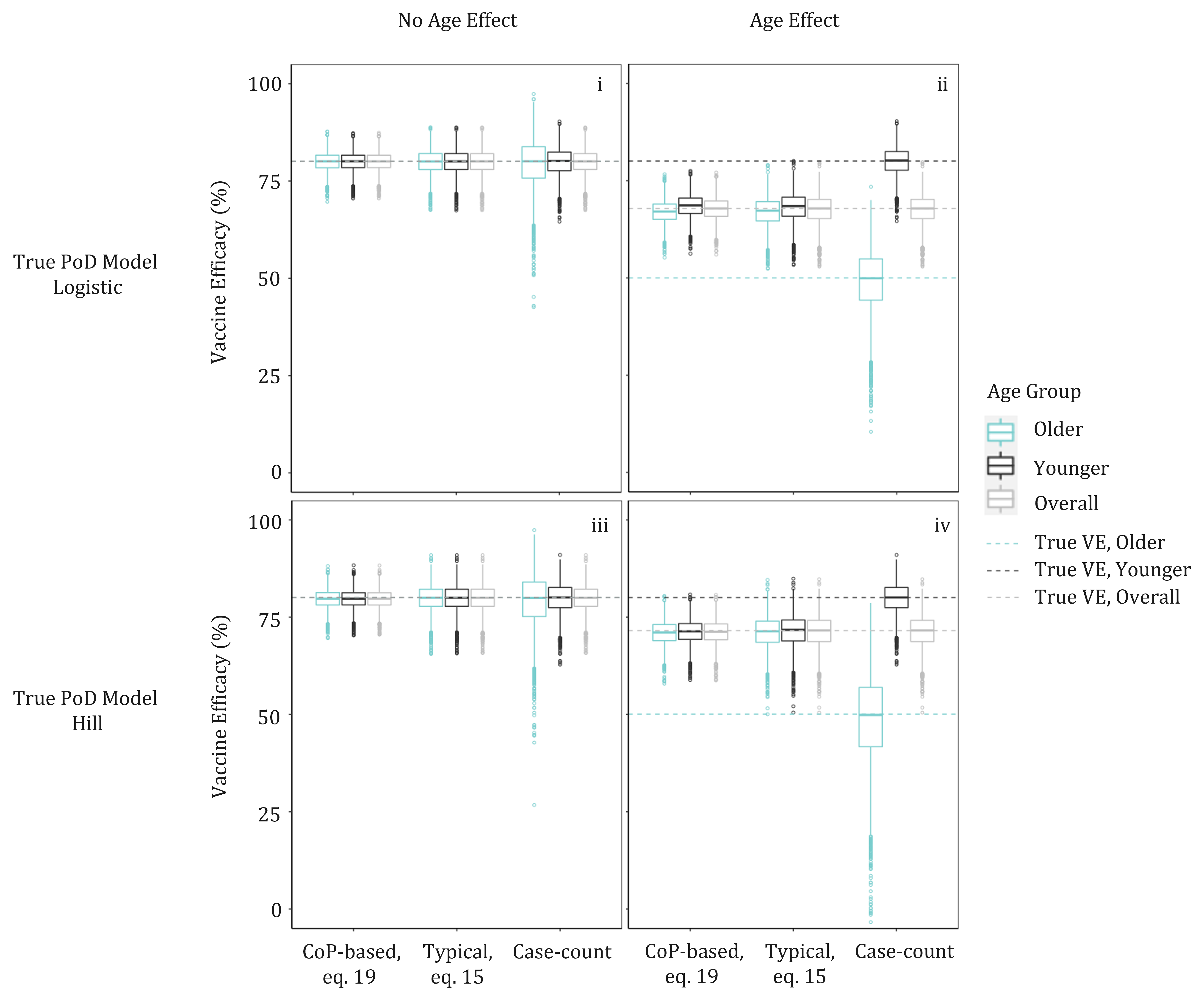
**

**Figure S5:** **Models 15 and 19 provide biased estimates of VE in some scenarios.**

Age-group-specific distributions of VE point estimates for each simulated scenario using CoP-based logistic regression (quadratic model without an interaction, Eq. 19, used for VE estimation), typical logistic regression (model without an interaction, Eq. 15, used for VE estimation), and case-counting. VE estimation by Eq. 19 is accurate in scenarios with no age effect (i, iii); VE in subgroups is biased if VE differs across subgroups (ii, iv). VE estimation by Eq. 15 is accurate in scenarios with no age effect (i, iii); VE in subgroups is biased if VE differs across subgroups (ii, iv). VE estimation by case-counting is accurate in all scenarios.

**
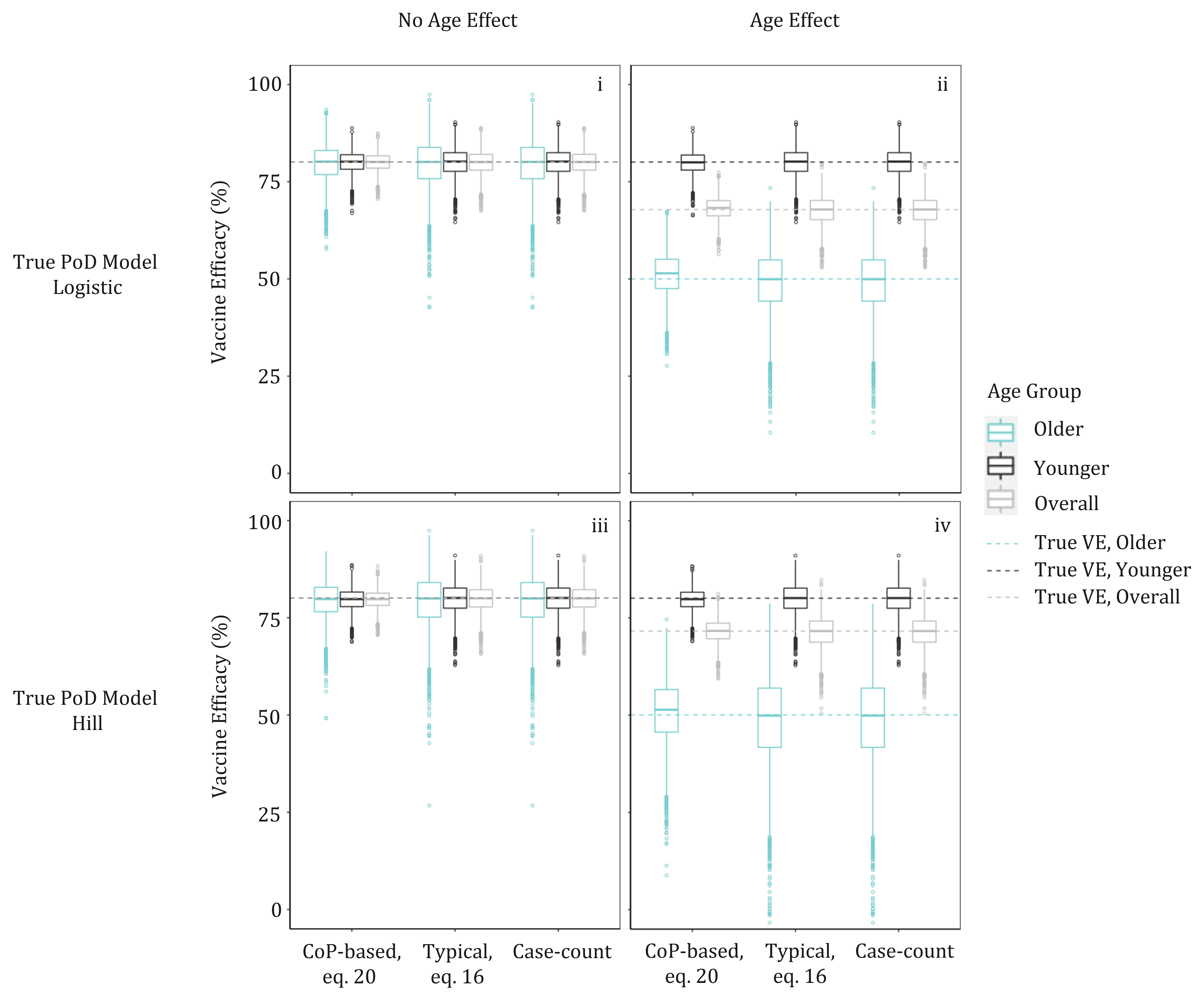
**

**Figure S6:** **Models 16 and 20 provide accurate (unbiased) estimates of VE in all scenarios.**

Age-group-specific distributions of VE point estimates for each simulated scenario using CoP-based logistic regression (quadratic model with an interaction, Eq. 20, used for VE calculation), typical logistic regression (model with an interaction, Eq. 16, used for VE calculation), and case-counting. The term “unbiased” is used because the median is within 2% of the true value in every scenario, so any difference is unlikely to be important (clinically or statistically). In scenarios ii and iv, the CoP-based estimate in older subgroup appear to be slightly biased, but the differences between the median value (51.4% for scenario ii, 51.4% for scenario iv) and the true (simulated) values (50.0% for scenario ii, 50.1% for scenario iv) are clinically insignificant. In every scenario and subgroup, the CoP-based approach provides the lowest mean squared error (Supplementary Material, Table S5).
